# Conformal hexagonal-boron nitride dielectric interface for tungsten diselenide devices with improved mobility and thermal dissipation

**DOI:** 10.1038/s41467-019-09016-0

**Published:** 2019-03-13

**Authors:** Donghua Liu, Xiaosong Chen, Yaping Yan, Zhongwei Zhang, Zhepeng Jin, Kongyang Yi, Cong Zhang, Yujie Zheng, Yao Wang, Jun Yang, Xiangfan Xu, Jie Chen, Yunhao Lu, Dapeng Wei, Andrew Thye Shen Wee, Dacheng Wei

**Affiliations:** 1 State Key Laboratory of Molecular Engineering of Polymers, Fudan University, Shanghai, 200433 China; 2 Department of Macromolecular Science, Fudan University, Shanghai, 200433 China; 3 Center for Phononics and Thermal Energy Science, School of Physics Science and Engineering, and Institute for Advanced Study, Tongji University, Shanghai, 200092 China; 4 China–EU Joint Lab for Nanophononics, Shanghai Key Laboratory of Special Artificial Microstructure Materials and Technology, School of Physics Science and Engineering, Tongji University, Shanghai, 200092 China; 5 Department of Physics, National University of Singapore, Singapore, 117542 Singapore; 6 International Center for New-Structured Materials and School of Materials Science and Engineering, Zhejiang University, Hangzhou, 310027 China; 7 Key Laboratory of Multi-scale Manufacturing Technology, Chongqing Institute of Green and Intelligent Technology, Chinese Academy of Sciences, Chongqing, 400714 China

## Abstract

Relatively low mobility and thermal conductance create challenges for application of tungsten diselenide (WSe_2_) in high performance devices. Dielectric interface is of extremely importance for improving carrier transport and heat spreading in a semiconductor device. Here, by near-equilibrium plasma-enhanced chemical vapour deposition, we realize catalyst-free growth of poly-crystalline two-dimensional hexagonal-boron nitride (2D-BN) with domains around 20~ 200 nm directly on SiO_2_/Si, quartz, sapphire, silicon or SiO_2_/Si with three-dimensional patterns at 300 °C. Owing to the atomically-clean van-der-Walls conformal interface and the fact that 2D-BN can better bridge the vibrational spectrum across the interface and protect interfacial heat conduction against substrate roughness, both improved performance and thermal dissipation of WSe_2_ field-effect transistor are realized with mobility around 56~ 121 cm^2^ V^−1^ s^−1^ and saturated power intensity up to 4.23 × 10^3^ W cm^−2^. Owing to its simplicity, conformal growth on three-dimensional surface, compatibility with microelectronic process, it has potential for application in future two-dimensional electronics.

## Abstract (web summary)

Plasma-enhanced chemical vapour deposition (PECVD) is an industrially compatible microelectronics technology. Here, the authors use PECVD to obtain low-temperature, catalyst-free growth of poly-crystalline two-dimensional hexagonal-boron nitride, thus enabling superior thermal dissipation in WSe_2_ field-effect transistors with mobility up to 121 cm^2^ V^−1^ s^−1^.

## Introduction

Tungsten diselenide (WSe_2_), as a two-dimensional (2D) semiconductor with one layer of W atoms sandwiched between two layers of Se atoms, has been regarded as one of the promising materials in future electronics, spin-electronics, and optoelectronics, owing to its excellent physical, optical, and electrical properties^1,2^. Whereas, the relatively low field-effect mobility is one of the main constraints preventing WSe_2_ from becoming a competing channel material for practical applications^3–5^. Till now, the room-temperature field-effect mobility of WSe_2_ grown by chemical vapor deposition (CVD) is normally around 30 cm^2^ V^−1^ s^−1^, lower than that required in high-performance electrical or photoelectrical devices^3–5^. Meanwhile, due to the ultra-low thermal conductance of WSe_2_ (0.05 W m^−1^ K^−1^)^6^, the requirement for efficient thermal dissipation is another significant factor in practical device application. In an electrical device, charge transport occurs at the interface between the semiconductor layer and the underlying substrate, where joule heating is generated. Thus, it is well known that the device mobility can be largely affected by charge impurities and roughness at the dielectric interface^7–15^. On the other side, the contact thermal interface is normally considered to be the bottleneck for efficient thermal dissipation rather than the material’s thermal conductivity itself^16–18^. Therefore, the dielectric interface is of great importance not only for improving the device mobility but also for removing heat from source. Till now, modifying the dielectric interface by self-assembled monolayers, bilayer polymeric dielectrics, plasma treatment, etc. has been developed to improve the device mobility^19^. To solve the interfacial thermal dissipation issue, some attempts such as covalent bonding or forming epitaxial interface have been made^20,21^, however practical applications of these approaches have had limited success due to the high cost, complicated modification process, lack of scalability, negative impact on the device mobility as well as poor compatibility with microelectronic processes. Till now, a simple, scalable, and compatible methodology to modify the dielectric interface for improving both the mobility and the thermal dissipation is still lacking, hampering recent efforts toward high-performance and stable electrical devices based on WSe_2_ or other 2D semiconductors.

Hexagonal-boron nitride (*h*-BN) has attracted much attention in recent years, as this material combines atomic-scale thickness with high dielectric constant (∼4), a wide bandgap, chemical inertness, excellent mechanical strength, and flexibility^9,14,22^. The in-plane thermal conductivity is up to ~200 to ~500 W m^−1^ K^−1^, several hundred times higher than that of amorphous SiO_2_ currently used in silicon-on-insulator device^23^. More importantly, 2D *h*-BN (2D-BN) has been normally regarded as a promising dielectric interface material for future electronics^9,14^. It has an atomically smooth surface without dangling bonds or charge traps, avoiding substrate surface roughness and carrier scattering from charge surface states or impurities. As a result, greatly improved carrier mobility of graphene (up to 100,000 cm^2^ V^−1^ s^−1^), black phosphorus, MoS_2_, organic crystals has been achieved on 2D-BN^9–11,14,15^, however its potential application in thermal dissipation at the semiconductor/dielectric interface is usually ignored. In the application, large-area 2D-BN needs to be placed on a desired surface. Current preparation methods require 2D-BN to be transferred from metals or solutions onto another surface for various applications^12,14,24–26^. The transfer process normally induces impurities, wrinkles, or breakage of the 2D-BN samples, which destroy the ideal van-der-Waals dielectric surface and cause interstices or incompact contact at the interface^12,14,24–26^, leading to the possibility of degraded performance or inefficient interfacial thermal dissipation for the WSe_2_ or other 2D semiconductor-based devices.

Although CVD on metal catalyst surface such as Cu, Ni, Pt, and Cu–Ni alloy can produce highly crystalline large-area 2D-BN^27–30^, it still requires the transfer process and a high growth temperature of 900–1000 °C. Recently, a few attempts on catalyst-free CVD have been made to grow 2D-BN directly on SiO_2_/Si^31^ or sapphire^32^. However, these processes require extremely high growth temperature above 1100 or 1400 °C, respectively. In an industrial-scale production, high temperature implies large energy consumption, high cost, and a decrease in compatibility with microelectronics fabrication processes. Plasma-enhanced CVD (PECVD) is a widely used industrial technology, which has high compatibility with current microelectronics fabrication process. To solve this problem, PECVD is particularly attractive, since the high energy plasma environment can decompose the precursor molecules at room temperature, thus enabling low-temperature growth of boron nitride (BN) materials directly on various surfaces^33–35^. Without metal catalysts, structural defects readily form at the edges and terminate the crystal growth. As a result, amorphous BN, disordered *h*-BN, or cubic-BN thick films are normally obtained by PECVD^33–35^, with a thickness about tens or hundreds of nanometers and a quality lower than that required for device applications. Till now, the catalyst-free growth of 2D-BN (mono-layered or few-layered) by PECVD is still absent.

Herein, we find that efficient crystal growth of 2D-BN takes place in a near-equilibrium state between the competition of etching and growth in PECVD. Based on this finding, we develop a near-equilibrium PECVD (ne-PECVD) to modify the dielectric interface, which realizes catalyst-free growth of uniform poly-crystalline 2D-BN with domains around 20–200 nm directly on SiO_2_/Si, quartz, sapphire, silicon, or even SiO_2_/Si with three-dimensional (3D) structures at a temperature as low as 300 °C, hundreds of degrees lower than that previously reported for 2D-BN growth. The thickness is precisely controlled from monolayer to four-layer by the growth time. After modification, the substrates have a smooth, atomically clean, and tightly contacted conformal van-der-Waals dielectric interface, which can be directly used to grow WSe_2_ by CVD (CVD-WSe_2_) for field-effect transistors (FETs). As a result, the device has an improved mobility around 56–121 cm^2^ V^−1^ s^−1^, higher than that on bare SiO_2_/Si (2–21 cm^2^ V^−1^ s^−1^) and the reported results of CVD-WSe_2_ FETs on SiO_2_/Si^3–5^, and exhibits high stability with increased saturated power density up to 4.23 × 10^3^ W cm^−2^. Both experimental and simulation results show that the conformal 2D-BN produced by ne-PECVD can better bridge the vibrational spectrum at the semiconductor/dielectric interface against substrate roughness. After modification, the interfacial thermal resistance of CVD-WSe_2_ on SiO_2_/Si decreases by 4.55 × 10^−8^ m^2^ K W^−1^ to a value smaller than 4.2 × 10^−8^ m^2^ K W^−1^, indicating an improved thermal dissipation at the dielectric interface.

## Results

### Growth of 2D-BN by ne-PECVD

**Fig. 1:**
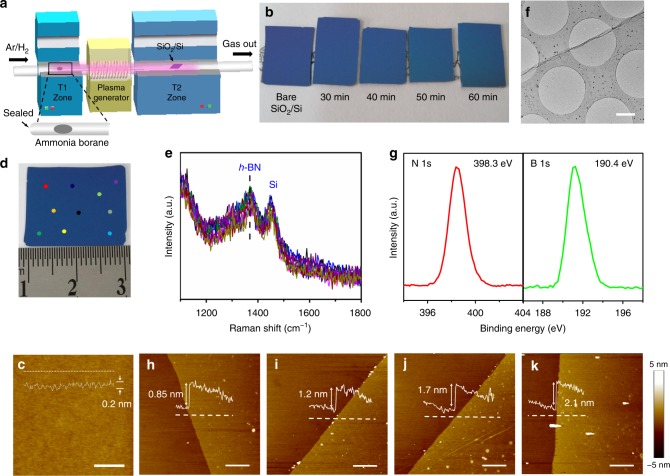
Growth of two-dimensional hexagonal-boron nitride (2D-BN) by near-equilibrium plasma-enhanced chemical vapor deposition (PECVD). **a** Schematic illustration of the PECVD system. **b** Optical image of bare SiO_2_/Si, 2D-BN grown on SiO_2_/Si after 30, 40, 50, and 60 min growth, respectively. **c** Atomic force microscope (AFM) and **d** optical image of 2D-BN grown on SiO_2_/Si after 30 min growth. **e** Raman spectra collected from the dots in **d**. **f** Transmission electron microscope (TEM) image of a 2D-BN transferred onto a TEM grid. **g** X-ray photoelectron spectroscopy N1*s* and B1*s* spectra of 2D-BN grown on SiO_2_/Si. **h**–**k** AFM images of 2D-BN transferred to other SiO_2_/Si. The growth time is 30, 40, 50, and 60 min, respectively. The scale bars are 500 nm in **c**, 1 μm in **f**, and 2 μm in **h**–**k**

The PECVD setup is shown in Fig. 1a, which is composed of a 2-inch quartz tube mounted inside two tubular furnaces and a radiofrequency (13.56 MHz) plasma generator between them. Solid ammonia borane was placed in the center of the tubular furnace (*T*1) upstream, and then was evaporated and diffused into the zone downstream by an Ar/H_2_ (100 sccm/10 sccm) carrier gas when the temperature of *T*1 rose to 110 °C. In the experiment, to obtain a steady precursor supply, ammonia borane was placed in a small quartz tube with one end sealed. 2D-BN films (Fig. 1b) were grown on SiO_2_/Si at 500 °C in 30 W plasma (860 mTorr) for 30, 40, 50, and 60 min in the center of the tubular furnace (*T*2) downstream. Figure 1c, d and Supplementary Fig. 1 show the atomic force microscope (AFM), optical, and scanning electron microscope (SEM) images of the as-grown 2D-BN (30 min) on SiO_2_/Si, respectively. The sample has a homogeneous color contrast and an ultra-smooth surface with roughness (around 0.2 nm) similar or even lower than that of bare SiO_2_/Si (Supplementary Fig. 2). Raman spectra (Fig. 1e) are collected from different locations of the sample, all of which have a homogeneous peak at ~1369 cm^−1^, corresponding to the *E*^2g^ phonon vibration of *h*-BN, indicating high uniformity of the as-grown film. The peak at ~1450 cm^−1^ is assigned to the third-order transverse optical phonon mode of Si of SiO_2_/Si substrate^29^. The *E*^2g^ band is located at a similar position to that of CVD 2D-BN (Supplementary Fig. 3), while the full width at half maxima (FWHM, ~49 cm^−1^) of the peak is larger than that (~24 cm^−1^) of CVD 2D-BN, revealing the poly-crystalline nature of the as-grown film with smaller grain size. After transferring the film to a carbon-copper grid, transmission electron microscope (TEM) image (Fig. 1f) shows a clean, continuous, uniform membrane-like structure. Cross-section TEM images (Supplementary Fig. 4) reveal layered crystalline structures, indicating the 2D nature of the material. The diffraction spot rings in the selected area electron diffraction (SAED) patterns (Supplementary Fig. 5a, 5b) indicate the poly-crystalline nature of the 2D-BN. With smaller electron beam spot size, six-fold symmetric diffraction spots (Supplementary Fig. 5c, 5d) can be observed, showing that the sample is composed by small crystalline *h*-BN domains. Energy-dispersive spectroscopy (EDS) collected from the membrane (Supplementary Fig. 6) exhibits B, N element peaks without any other peaks except C, Cu, and O (from carbon-copper grid), indicating the high sample purity. X-ray photoelectron spectroscopy (XPS, Fig. 1g) reveals an almost equal composition of B and N elements (1:1.12). Symmetrical B 1*s* and N 1*s* peaks are located at 190.4 and 398.3 eV, respectively, indicating that the as-grown sample is predominantly composed of B–N bonds with *sp*^2^ hybridization^31^. To measure the thickness, the as-grown samples with continuous areas up to several square centimeters are transferred to another SiO_2_/Si substrate using poly-methyl-methacrylate (PMMA). The thicknesses (Fig. 1h–k, Supplementary Fig. 7) measured from the boundaries between the sample and SiO_2_/Si are 0.85 nm (30 min), 1.2 nm (40 min), 1.6 nm (50 min), and 2.1 nm (60 min), which correspond to 1, 2, 3, and 4 atomic layers of 2D-BN excluding the roughness of SiO_2_/Si^30^. The thickness shows a good correlation with the growth time (Supplementary Fig. 8), in consistence with the cross-section TEM results (Supplementary Fig. 4). Therefore, large-area uniform 2D-BN films are grown on SiO_2_/Si with controllable thickness from monolayer to multilayers (Supplementary Figs 4, 9).

**Fig. 2:**
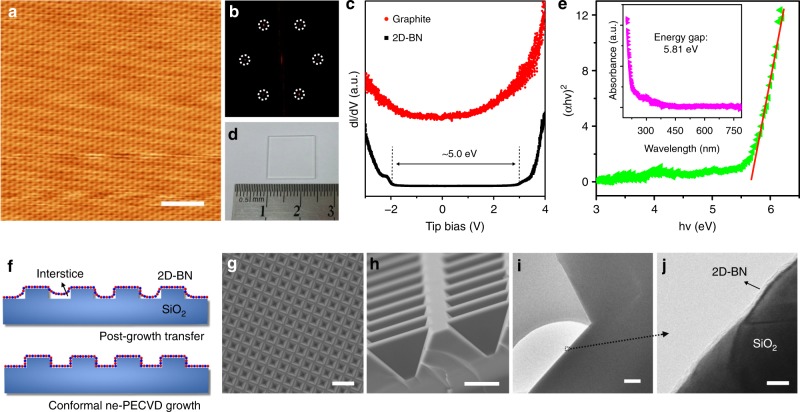
Characterization of two-dimensional hexagonal-boron nitride (2D-BN). **a** Scanning tunneling microscopy image (*V*_Tip_ = −0.5 V) and **b** fast Fourier transform (FFT) image of 2D-BN on highly oriented pyrolytic graphite (HOPG). The dashed circles highlight the points of the FFT pattern. **c** Scanning tunneling spectroscopy spectra collected from 2D-BN and HOPG (set point: *V*_Tip_ = 3.0 V, *I*_Tip_ = 48 pA). **d** Optical image and **e** Tauc plot (inset: ultraviolet spectrum) of 2D-BN grown on quartz. **f** Schematic illustration, **g** top view scanning electron microscope (SEM), **h** side view SEM, and **i** cross-section transmission electron microscope (TEM) image of 2D-BN grown by near-equilibrium plasma-enhanced chemical vapor deposition (ne-PECVD) on SiO_2_/Si with three-dimensional structures. **j** The higher magnified TEM image of the areas indicated in **i** by a dashed frame. The scale bars are 2 nm in **a**, 10 μm in **g**, 2 μm in **h**, 200 nm in **i**, and 5 nm in **g**

The samples are transferred to highly oriented pyrolytic graphite (HOPG) for scanning tunneling microscopy (STM) studies. The grain size is measured in the range of 20 nm to more than 200 nm (Supplementary Fig. 10). Magnified low-temperature STM (LT-STM) image (Fig. 2a) reveals a honeycomb-like crystalline structure with a nearly atomically clean surface, while the fast Fourier transform (Fig. 2b, Supplementary Fig. 11) image confirms the hexagonal lattice structure. Moiré patterns (Supplementary Fig. 12), which are attributed to the lattice mismatching and rotation of the 2D-BN on HOPG, are observed, indicating high crystallinity of the 2D-BN domains. The scanning tunneling spectroscopy (STS, Fig. 2c) curve collected from HOPG is a typical d*I*/d*V* of graphite with no bandgap, while the density of states of 2D-BN is remarkably depressed near the Fermi level with a measured band gap around 5.0 eV. Compared with intrinsic 2D-BN (5.9 eV), the reduced bandgap is attributed to the weak interaction and the screening from the HOPG substrate^36,37^. The bandgap can also be measured by ultraviolet-visible (UV-vis) absorption spectroscopy. UV-vis spectrum (Fig. 2e) of the 2D-BN grown on quartz (Fig. 2d) exhibits zero absorbance in the visible-light region and a sharp peak at ~200 nm, consistent with the results reported previously^27^. The optical energy gap is calculated by Tauc’s equation: *αhν* = *A*(*hν* − *E*_g_)^1/2^, where *α* is the optical absorption coefficient, *hν* is the energy of incident photon, *A* is the proportionality constant, and *E*_g_ is energy gap^27^. The (*αhν*)^2^ vs. *hν* curve acquired from the sample is shown in Fig. 2e, and the calculated optical energy gap is 5.81 eV, which is larger than that of bulk *h*-BN and close to intrinsic 2D-BN^27^.

To confirm the conformal growth of 2D-BN, SiO_2_/Si with 3D structures (Fig. 2f) were used as the growth substrate. After ne-PECVD, XPS spectra (Supplementary Fig. 13) indicate the growth of 2D-BN, while SEM images (Fig. 2g, h, Supplementary Fig. 14) show a clean 3D surface without any bubbles, wrinkles, or incompact contacts, which normally exist in the post-growth transfer CVD samples (Supplementary Fig. 15). The 3D structures were cut and transferred to a copper grad. Cross-section TEM images (Fig. 2i, j) show a closely contacted layer of 2D-BN on the 3D surface, indicating the conformal growth and the potential of ne-PECVD in modifying 3D dielectric interface for devices with 3D configuration. Besides SiO_2_/Si, substrates like sapphire, silicon, quartz (Supplementary Fig. 16) can also be used in ne-PECVD, and the growth temperature on SiO_2_/Si can decrease to as low as 300 °C (Supplementary Fig. 17), hundreds of degrees lower than that reported previously^27–32^.

### Near-equilibrium growth mechanism

**Fig. 3:**
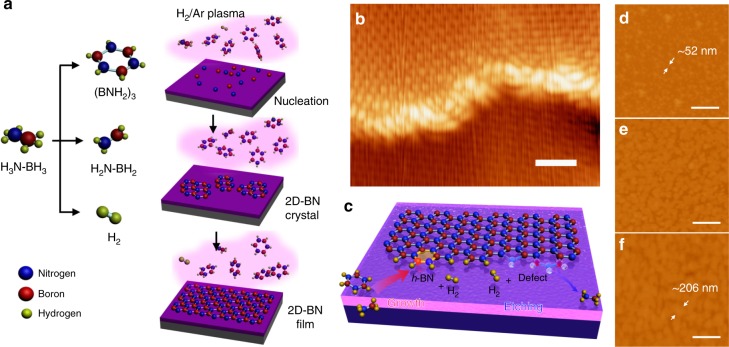
Growth mechanism of near-equilibrium plasma-enhanced chemical vapor deposition (ne-PECVD). **a** Schematic illustration of the growth process of two-dimensional hexagonal-boron nitride (2D-BN) on SiO_2_/Si. **b** Scanning tunneling microscopy image of a 2D-BN edge (*V*_Tip_ = −0.5 V). **c** Schematic illustration of the competition process of 2D-BN growth and etching in ne-PECVD. **d**–**f** Atomic force microscope images of the 2D-BN domains grown on SiO2/Si by ne-PECVD for **d** 8 min, **e** 15 min, and **f** 20 min. The scale bars are 1 nm in **b** and 500 nm in **d**–**f**

In the growth, ammonia borane is evaporated (H_2_, monomeric aminoborane, borazine also exist owing to thermal decomposition)^38^, and then decomposed into boron and nitrogen species (radicals, ions, and atoms) by the plasma. These highly reactive species overcome the large threshold barrier required, leading to catalyst-free growth of BN materials directly on the inert surface at low temperature. However, these species are inclined to form structural defects on edges, which prevent the crystal growth of 2D-BN. Some literatures have demonstrated the etching of BN materials by H_2_, Ar, O_2_, or H_2_/Ar plasma^39–41^, thus a competition process of etching and nucleation/deposition exists in the PECVD process^42^, however the 2D-BN lattice is energetically highly stable. Experiments show no obvious etching of 2D-BN in H_2_/Ar plasma even after 60 min treatment (Supplementary Fig. 18). Thus, in normal cases, the nucleation/deposition dominates, and disordered or amorphous BN films are grown in a non-equilibrium state as shown in Supplementary Fig. 19, 20^33–35^. Nevertheless, the literature and first-principle calculation (Supplementary Note 1) show that the edge defect has a higher energy^43^, compared with pristine *h*-BN lattice and H-passivated edges. The energy increases by 7.458 and 9.526 eV, when a B_3_N_2_ or B_2_N_3_ pentagonal defect forms on H-passivated armchair edges (Supplementary Fig. 21). Thus, the etching tends to occur at the edge defects. Owing to the low etching rate, a slow and steady precursor feeding is required to establish a reversible competition between the nucleation/growth and the etching. Therefore, controlled experiments (Supplementary Fig. 22, 23) show that a slow and steady precursor feeding is pivotal for the 2D-BN growth, which requires placing the precursor in a small quartz tube with one end sealed and maintains at a temperature (*T*1) below 115 °C. In such a near-equilibrium state (Supplementary Note 2), moderate etching by the H_2_/Ar plasma removes defects generated on the edges and keeps the edges atomically smooth (Fig. 3b) and active during the whole PECVD process, resulting in efficient crystal growth of 2D-BN directly on the inert surface without any catalyst (Fig. 3c). The 2D-BN crystals nucleate and the grain sizes gradually increase up to more than 200 nm (Fig. 3d–f), and finally a continuous 2D-BN film is obtained on the substrate.

### CVD growth of WSe_2_ on 2D-BN

**Fig. 4:**
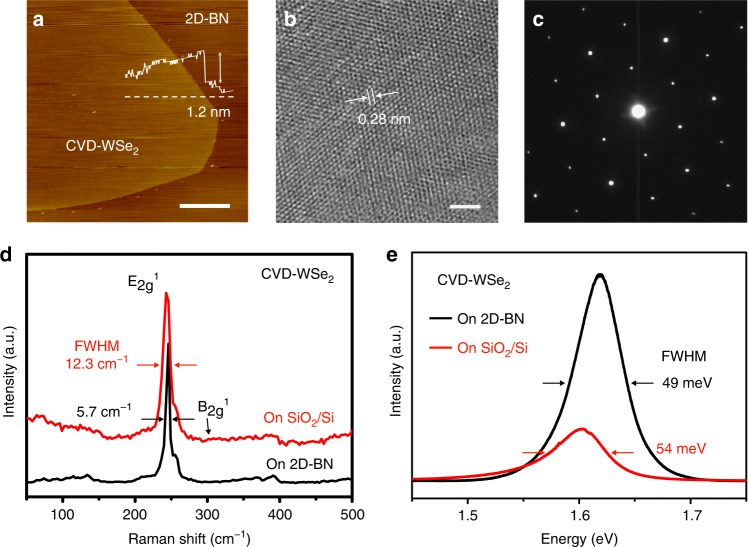
Direct growth of CVD-WSe_2_ on two-dimensional hexagonal-boron nitride (2D-BN). **a** Atomic force microscope image of a CVD-WSe_2_ crystal grown on 2D-BN. **b** High-resolution transmission electron microscope image and **c** selected area electron diffraction patterns of a CVD-WSe_2_ crystal. The (100) plane space is 0.28 nm. **d** Raman spectra of a CVD-WSe_2_ crystal grown on 2D-BN or on bare SiO_2_/Si. The full width at half maxima (FWHM) of the *E*^1^_2g_ band is 5.7 and 12.3 cm^−1^, respectively. **e** Photoluminescence spectra of the CVD-WSe_2_ grown on 2D-BN or on bare SiO_2_/Si. The FWHM of the peak is 49 and 54 meV, respectively. The scale bars are 5 μm in **a** and 2 nm in **b**

The benefit of ne-PECVD is that it modifies flat or 3D substrates with an atomically clean conformal van-der-Waals dielectric surface, which can be directly used in electrical devices. We grew 2D WSe_2_ crystals (CVD-WSe_2_) on 2D-BN/SiO_2_/Si by CVD for back-gated FETs. In the growth, a quartz boat with 450 mg Se powder was placed upstream in the furnace, while 130 mg WO_3_ powder was placed downstream (Supplementary Fig. 24). The substrates were placed on the top of WO_3_ with the surface facing down. The upstream and downstream zones were gradually heated from room temperature to 300 and 920 °C in 300 sccm pure Ar within 30 min. And then, a mixture of 85 sccm Ar and 15 sccm H_2_ was introduced into the furnace. After 15 min growth, the furnace was naturally cooled down to 300 °C, and was fast cooled down to room temperature. AFM and optical images (Fig. 4a, Supplementary Fig. 25) show that most of the as-grown CVD-WSe_2_ crystals have a triangular or hexagonal shape with a size of about 30–200 μm and a height of about 1.2 nm. The high-resolution TEM image and the hexagonal arrangement of the SAED pattern (Fig. 4b, c) show the CVD-WSe_2_ sample is well crystallized with the measured lattice spacing of 0.28 nm, in consistent with the (100) plane spacing of 2H-WSe_2_. In Raman spectrum (Fig. 4d), an intrinsic *E*^1^_2g_ band at 248 cm^−1^ and the absence of the *B*^1^_2g_ mode at 307 cm^−1^ indicate the monolayer nature of the CVD-WSe_2_^7,25,44^. The photoluminescence (PL) spectrum (Fig. 4e) exhibits strong emission at 768 nm, corresponding to the direct bandgap of monolayer WSe_2_^7,8,24,25^. The highly crystalline structure (Fig. 4b), as well as the narrow *E*^1^_2g_ Raman peak (a FWHM of 5.7 cm^−1^) and the sharp PL emission (a FWHM of 49 meV) without the defect emission at 1.54 eV^7,8,25,44^, indicate high quality of the CVD-WSe_2_ samples grown on the ne-PECVD 2D-BN.

### Enhanced mobility of WSe_2_ on 2D-BN

**Fig. 5:**
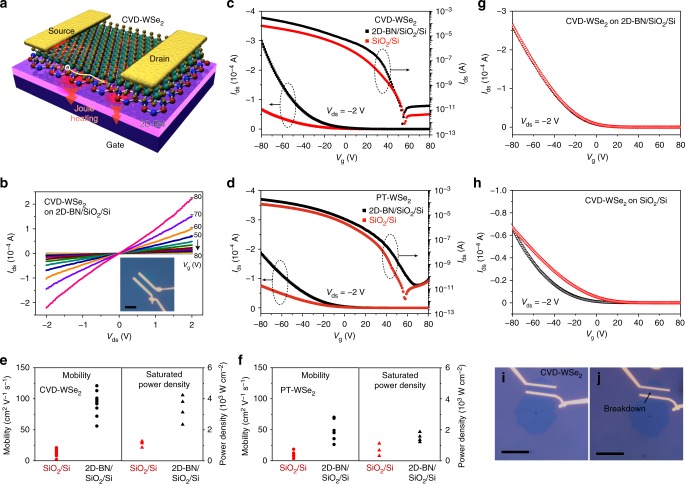
Two-dimensional hexagonal-boron nitride (2D-BN) as dielectric interfacial material in field-effect transistors (FETs). **a** Schematic illustration of a CVD-WSe_2_ FET device using 2D-BN as the dielectric interface. **b** Output curves of the CVD-WSe_2_ FET on 2D-BN/SiO_2_/Si. The inset shows the optical image of the device. **c**, **d** Transfer curves (*V*_ds_ = −2 V) of the CVD-WSe_2_ FET and a PT-WSe_2_ FET on 2D-BN/SiO_2_/Si and on bare SiO_2_/Si. **e**, **f** Mobility and saturated power density of the CVD-WSe_2_ FET and the PT-WSe_2_ FET on 2D-BN/SiO_2_/Si and on bare SiO_2_/Si. **g**, **h** Transfer curve (*V*_ds_ = −2 V) of a CVD-WSe_2_ FET on 2D-BN/SiO_2_/Si and on SiO2/Si. The black and red curves are obtained when *V*_g_ sweeps from 80 to −80 V and from −80 to 80 V, respectively. **i**, **j** Optical images of a CVD-WSe_2_ FET on 2D-BN/SiO_2_/Si before and after current breakdown. Scale bars in **b**, **i**, **j** are 20 μm

After CVD growth, the back-gated FETs were directly fabricated by using 2D-BN/SiO_2_/Si as gate dielectric and back gate, using CVD-WSe_2_ as the conducting channel (Fig. 5). All devices were current-annealed or thermal-annealed, and then were measured under ambient conditions. As a comparison, CVD-WSe_2_ FETs were also fabricated using bare SiO_2_/Si. Drain-source current (*I*_ds_) vs. drain-source voltage (*V*_ds_) (output curve, Fig. 5b) and *I*_ds_ vs. gate voltage (*V*_g_) (transfer curve, *V*_ds_ at −2 V, Fig. 5c) show a typical *p*-type characteristic with an on/off ratio up to 1 × 10^8^ and 1 × 10^7^ for CVD-WSe_2_ FETs on 2D-BN/SiO_2_/Si and on bare SiO_2_/Si substrates, respectively, when the gate voltage sweeps from −80 to 80 V. The mobility is calculated (Supplementary Note 3, Supplementary Fig. 26) by the equation^3–5^:

$$\mu = (\frac{L}{{WC_{\mathrm{i}}V_{\mathrm{ds}}}})(\frac{{\Delta I_{\mathrm{ds}}}}{{\Delta V_{\mathrm{g}}}})$$

where *C*_i_ is the dielectric capacitance, *W* is the channel width, *L* is the channel length, and (Δ*I*_ds_/Δ*V*_g_) is the slope of the transfer curves in the linear regime. The calculated mobility of CVD-WSe_2_ on 2D-BN/SiO_2_/Si is around 56–121 cm^2^ V^−1^ s^−1^, which is higher than that of CVD-WSe_2_ grown on bare SiO_2_/Si (Fig. 5e, 2–21 cm^2^ V^−1^ s^−1^) and the room-temperature mobility (10–30 cm^2^ V^−1^ s^−1^) of monolayer WSe_2_ on bare SiO_2_/Si or Al_2_O_3_ reported previously^3–5^. The increased mobility is in good agreement with that of 2D semiconductor devices on peel-off or CVD 2D-BN reported previously^9,12–15^, indicating the high quality of the 2D-BN produced by ne-PECVD.

There are several reasons for the increased mobility of CVD-WSe_2_ on ne-PECVD 2D-BN. The main reason is the smooth van-der-Waals conformal surface of the 2D-BN, as shown in STM image (Fig. 2a). Atomically thin 2D materials have a large surface-to-volume ratio, thus the carrier conduction is significantly influenced by the dielectric interface. At the interface, the modification with 2D-BN avoids surface roughness, dangling bonds, surface charge impurities or traps, which normally exist on bare SiO_2_/Si^7–15^, and decreases charge carrier scattering. As a result, the 2D-BN remarkably improves the carrier mobility of 2D or organic materials^7–15,25^. For instance, Kim et al. reported that MoS_2_ mobility increased from 10 to 42 cm^2^ V^−1^ s^−1^ by introducing highly crystalline CVD *h*-BN between MoS_2_ and SiO_2_/Si^25^. In the case of CVD-WSe_2_ on ne-PECVD 2D-BN (Fig. 4e), the narrow symmetrical PL peak originated from the neutral exciton emission is observed^7,24,25^, while the PL of CVD-WSe_2_ on SiO_2_/Si is weaker with a red shift (17 meV) and unsymmetrical profile, corresponding to a charged exciton (trion) emission^7,24,25^. The PL spectra indicate that the ne-PECVD 2D-BN is more charge-neutral, which strongly suppresses and screens out the influence of charge impurities existing on bare SiO_2_/Si^7,24,25^. Moreover, the inert 2D-BN surface has low charge trap density. Joo et al. observed the reduction of interfacial traps density by 100 times compared with that on SiO_2_/Si^11^. To clarify trap states on 2D-BN, we investigated the hysteresis in the transfer characteristics of CVD-WSe_2_ FETs (Fig. 5g, h, Supplementary Fig. 27). On bare SiO_2_/Si or disordered PECVD BN film, an obvious hysteresis exists, while it becomes much smaller on ne-PECVD 2D-BN. This result reveals a charge trap-free dielectric interface of the ne-PECVD 2D-BN^10,11,14,15^, thus it avoids accumulation of charge impurities at the interface of SiO_2_/Si, which are normally regarded as carrier scattering centers to decrease the mobility^10,11,14,15^.

The other reason is the clean dielectric interface. Moderate etching effect of Ar/H_2_ plasma in ne-PECVD removes the surface impurities generated on 2D-BN, resulting in an atomically clean surface (Fig. 2a). After ne-PECVD, CVD-WSe_2_ crystals are directly grown on 2D-BN/SiO_2_/Si, avoiding post-growth transfer. The post-growth transfer normally involves a deposition of polymers and a solution-washing process, thus this process introduces contamination or defects at the interface and causes low quality of interlayer contact^8,26,45^, leading to reduction of the device performance^7,8,45^. Control experiments show that the post-growth transferred CVD-WSe_2_ (PT-WSe_2_) on 2D-BN/SiO_2_/Si has an obvious hysteresis in the transfer curve (Supplementary Fig. 27). The mobility (Fig. 5e, f) decreases to 26.3–70.2 cm^2^ V^−1^s^−1^, lower than that (56–121 cm^2^ V^−1^s^−1^) of the directly grown samples. Therefore, the moderate plasma etching as well as the direct CVD growth, in principle, realizes a clean and smooth interlayer interface between 2D-BN and WSe_2_, compared with the transferred structures, which is one of the key factors to obtain the intrinsic properties of WSe_2_ or other 2D materials^7,26,46,47^.

Moreover, the improved sample quality, when using 2D-BN as the growth substrate^2,24,48^, should be one of other probable reasons for the increased mobility of CVD-WSe_2_ on ne-PECVD 2D-BN. Uchida et al. and Okada et al. show that higher quality of transition metal dichalcogenides (TMDs) such as WS_2_ can be produced on flat 2D-BN without dangling bonds and contaminations compared with the sample grown on SiO_2_ or post-growth transferred 2D-BN^2,24^. Similarly, in this work, the FWHM of the Raman *E*^1^_2g_ peak (Fig. 4d) and the FWHM of the PL peak (Fig. 4e) decreases from 12.3 cm^−1^ and 54 meV (grown on SiO_2_/Si) to 5.7 cm^−1^ and 49 meV (grown on 2D-BN), respectively, indicating that higher crystallinity of the CVD-WSe_2_ is obtained when the growth takes place on ne-PECVD 2D-BN^2,24,44^. In order to avoid the difference in sample quality, we grew CVD-WSe_2_ on SiO_2_/Si and then transferred to other substrates (Fig. 5d–f). Although the mobility of PT-WSe_2_ on 2D-BN/SiO_2_/Si (26.3–70.2 cm^2^ V^−1^ s^−1^) is lower than that of CVD-WSe_2_ on 2D-BN/SiO_2_/Si due to the impurities or defects introduced by the post-growth transfer process^7,26,46,47^, it is still higher than that of PT-WSe_2_ on bare SiO_2_/Si (3.8–18.6 cm^2^ V^−1^ s^−1^), indicating that the improved WSe_2_ mobility on 2D-BN/SiO_2_/Si mainly originates from the 2D-BN layer rather than the sample quality.

### Improved thermal dissipation of WSe_2_ FETs on 2D-BN

The thermal dissipation is a great challenge for devices or integrated circuits with ultrahigh operating frequency, especially for WSe_2_ FETs, as the WSe_2_ is normally regarded as a material with the lowest thermal conductivity^6^. Although much research has demonstrated the 2D-BN as an ideal dielectric material for improving the device mobility, its potential application in improving the thermal dissipation of a FET device is usually ignored. We measured the saturated power density of CVD-WSe_2_ FETs on different substrates when current breakdown took place (Fig. 5i, j, Supplementary Fig. 28). The saturated power density (Fig. 5e, f), calculated by the power at the current breakdown divided by the device area, reaches up to 4.23 × 10^3^ (CVD-WSe_2_) and 1.87 × 10^3^ W cm^−2^ (PT-WSe_2_) on 2D-BN/SiO_2_/Si, higher than that on bare SiO_2_/Si, indicating that the ne-PECVD 2D-BN not only increases the device mobility but also increases device stability with higher saturated power density.

**Fig. 6:**
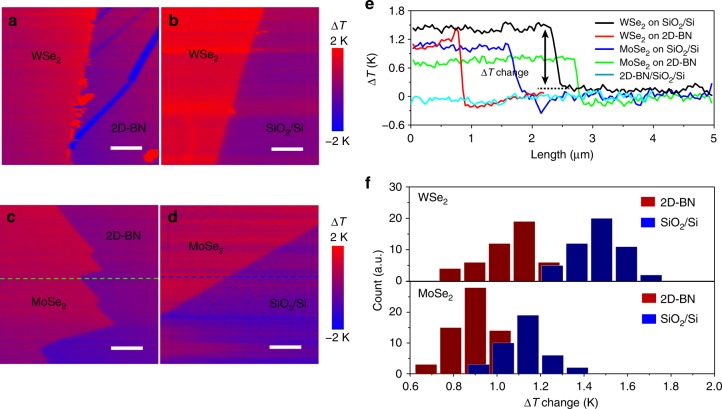
Scanning thermal microscope (SThM) measurement of the interfacial thermal dissipation. **a**–**d** SThM thermal images of **a** WSe_2_ on 2D-BN/SiO_2_/Si, **b** WSe_2_ on SiO_2_/Si, **c** MoSe_2_ on 2D-BN/SiO_2_/Si, and **d** MoSe_2_ on SiO_2_/Si. **e** The cross-sectional profiles where temperature changes (∆*T*) are recorded across the 2D-BN/SiO_2_/Si surface or the WSe_2_, MoSe_2_ edges along the dashed lines in **a**–**d**. **f** The histograms show the ∆*T* change across the WSe_2_ or MoSe_2_ edge on 2D-BN/SiO_2_/Si or SiO_2_/Si. Scale bars in **a**–**d** are 1 μm

The increased saturated power density is attributed to the improved interfacial thermal dissipation at the dielectric interface with 2D-BN. Different from thermal conductivity, interfacial thermal conductivity is a measure of an interface’s conductivity to thermal flow, which exists even at atomically perfect interfaces. Despite the large in-plane thermal conductivity of 2D-BN, it is atomically thin, the in-plane thermal flow can be ignored, and the interfacial thermal resistance across the dielectric interface dominates the thermal conduction^18,49^, which plays a critical role in the saturated power density of the devices. A scanning thermal microscope (SThM), which operates by scanning a sample solid surface with a sharp temperature-sensing tip, is a powerful tool for imaging sub-micron heat transfer at surface and subsurface levels^50^. When the tip scans on the sample surface by an active and contact mode; 2D-mapping thermal images of CVD-WSe_2_/2D-BN/SiO_2_ and CVD-WSe_2_/SiO_2_ (Fig. 6a, b) are obtained by monitoring the temperature changes (∆*T*) of the tip. As a comparison, other TMDs materials (CVD-MoSe_2_, Fig. 6c, d) are also measured. No obvious ∆*T* change is observed on 2D-BN/SiO_2_, owing to efficient thermal dissipation between the tip and the substrate across the 2D-BN. Although the sample (CVD-MoSe_2_ or CVD-WSe_2_)/2D-BN/SiO_2_ has more interfaces compared with the sample/SiO_2_, the former has lower ∆*T* change (Fig. 6e, f), indicating that the 2D-BN layer helps thermal dissipation from tip to substrate across the sample/dielectric interface (Supplementary Note 4).

**Fig. 7:**
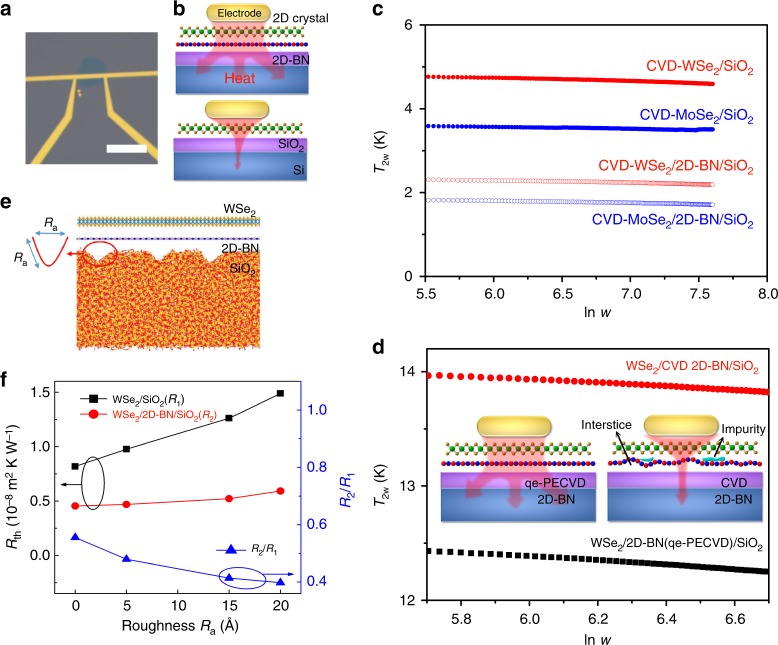
Differential 3*ω* measurement and molecular dynamics (MD) simulation of the interfacial thermal resistance. **a** Optical image of a CVD-WSe_2_ device for differential 3*ω* measurement. **b** Schematic images of the devices for 3*ω* measurement, in which Au/Cr is used as both the Joule heat source (current with a frequency of *w*) and the detection electrode (electrical signal with a frequency of 3*w*). **c** *T*_2*ω*_ vs. ln *ω* for CVD-WSe_2_/SiO_2_ (red solid), CVD-WSe_2_/2D-BN/SiO_2_ (red open), CVD-MoSe_2_/SiO_2_ (blue solid), and CVD-MoSe_2_/2D-BN/SiO_2_ (blue open) interfaces. **d** *T*_2*ω*_ vs. ln *ω* for WSe_2_/2D-BN/SiO_2_ interface. The 2D-BN/SiO_2_ substrate was prepared by ne-PECVD (black) or transferring CVD 2D-BN on SiO_2_/Si (red). **e** WSe_2_/2D-BN/SiO_2_ hybrid system used in MD simulation. **f** The effect of the substrate roughness (*R*_a_) on the interfacial thermal resistance (*R*_th_) for WSe_2_/SiO_2_ and WSe_2_/2D-BN/SiO_2_ hybrid systems from MD simulation. The scale bar in **a** is 20 μm

To quantify the thermal dissipation, interfacial thermal resistance measurements were carried out by differential 3*ω* method (Fig. 7a, b, Supplementary Fig. 29, Supplementary Note 5)^18,49^. Although the channel/2D-BN/SiO_2_ contains two interfaces, i.e. the channel/2D-BN interface and the 2D-BN/SiO_2_ interface, while the channel/SiO_2_ contains only one interface, all results, including CVD-WSe_2_/2D-BN/SiO_2_, PT-WSe_2_/2D-BN/SiO_2_, and CVD-MoSe_2_/2D-BN/SiO_2_ (Supplementary Fig. 30), exhibit an improved thermal dissipation in the case of the channel/2D-BN/SiO_2_. Compared with that of CVD-WSe_2_/SiO_2_ and CVD-MoSe_2_/SiO_2_, the interfacial thermal resistance of CVD-WSe_2_/2D-BN/SiO_2_ (Fig. 7a) and CVD-MoSe_2_/2D-BN/SiO_2_ (Supplementary Fig. 31) reduces by (4.55 ± 0.25) × 10^−8^ and (1.21 ± 0.20) × 10^−7^ m^2^ K W^−1^, respectively (Fig. 7c). In the case of the WSe_2_, to avoid the difference in sample quality on different growth substrates^48^, we measured PT-WSe_2_/2D-BN/SiO_2_ and PT-WSe_2_/SiO_2_ by differential 3*ω* method. The measurement shows reduced interfacial thermal resistance of PT-WSe_2_/2D-BN/SiO_2_ by (1.2 ± 0.20) × 10^−8^ m^2^ K W^−1^ compared with PT-WSe_2_/SiO_2_ (Supplementary Fig. 32). The calculated thermal resistance (4.2 × 10^−8^ or 1.1 × 10^−7^ m^2^ K W^−1^) by differential 3*ω* method corresponds to the sum of the substrate thermal resistance and the interfacial thermal resistance of CVD-WSe_2_/2D-BN/SiO_2_ or CVD-MoSe_2_/2D-BN/SiO_2_, respectively. Thus, the interfacial thermal resistance of CVD-WSe_2_/2D-BN/SiO_2_ and CVD-MoSe_2_/2D-BN/SiO_2_ is lower than 4.2 × 10^−8^ and 1.1 × 10^−7^ m^2^ K W^−1^ (Supplementary Note 5), showing better thermal dissipation compared with the results of graphene/SiO_2_ (1.24–5.56 × 10^−8^ m^2^ K W^−1^)^49^, MoSe_2_/Au/SiO_2_ (100–1000 × 10^−8^ m^2^ K W^−1^)^51^, etc.

Moreover, one benefit of ne-PECVD is that the conformal 2D-BN avoids impurities, interstices, or incompact contacts, which normally exist in post-growth transferred 2D-BN produced by CVD. The direct CVD growth of WSe_2_ on 2D-BN provides strong interlayer coupling^46^. As a result of the clean and tightly contacted interface, the differential 3*ω* measurement (Fig. 7d) shows that the interfacial thermal resistance of PT-WSe_2_/2D-BN (prepared by ne-PECVD)/SiO_2_ decreased by 1 × 10^−7^ m^2^ K W^−1^, compared with PT-WSe_2_/2D-BN (post-growth transferred CVD 2D-BN)/SiO_2_, indicating the significance of ne-PECVD 2D-BN with conformal van-der-Walls interface in device thermal management.

### Molecular dynamics simulation of interfacial thermal dissipation

The discrepancy in thermal dissipation should be related to the roughness of the interface that an additional layer of 2D-BN could help the WSe_2_ (or MoSe_2_) to conform onto the SiO_2_ surface, resulting in a reduced roughness and interface thermal resistance^18,49^. To clarify the mechanism, we compare the interfacial thermal resistance for WSe_2_/SiO_2_ (*R*1) and WSe_2_/2D-BN/SiO_2_ (*R*2) under different substrate surface roughness conditions by molecular dynamics (MD) simulations (Supplementary Note 6, Supplementary Fig. 33). Because of the broadened vibrational frequency distribution^52^, the 2D-BN layer works as an external material that can better fit the vibrational spectrum between SiO_2_ substrate and WSe_2_ layer. Therefore, the insertion of 2D-BN layer can reduce the interfacial thermal resistance, regardless of the substrate roughness. In the MD simulations, the size of the amorphous SiO_2_ substrate is 12.4 nm × 12.4 nm × 4 nm, and the monolayer 2D-BN and WSe_2_ conformally cover the substrate. In order to simulate the practical surface roughness of SiO_2_ substrate, we randomly removed surface atoms to form holes with a diameter of *R*_*a*_, as shown in Fig. 7e. For the smooth substrate, *R*1 is reduced by almost 50% after inserting a 2D-BN layer. More interestingly, *R*1 increases rapidly with the substrate roughness as a result of the enhanced rough surface scattering, while the interfacial heat conduction is protected by the 2D-BN layer against the substrate roughness, leading to the almost constant value of *R*2. As a result, the improvement in the interfacial heat conduction via the insertion of 2D-BN layer is even more pronounced at larger surface roughness (Fig. 7f, Supplementary Fig. 34). With 2 nm substrate roughness, *R*1 is reduced by 60%, which agrees well with our experimental results.

## Discussion

In this article, we directly modify the dielectric interface with poly-crystalline mono-/few-layer 2D-BN with domain size around 20–200 nm at a temperature as low as 300 °C. To the best of our knowledge, it is the lowest reported temperature for growing 2D-BN. The conformal growth on 3D surface is important for microelectronics manufacturing, however it is still difficult to be realized by existing 2D-BN preparation methods. Using ne-PECVD, large-area 2D-BN with desired thickness is directly grown on not only flat but also 3D inert surface without using metal catalyst. This method avoids the post-growth transfer process required in normal CVD process, forming a clean conformal van-der-Waals dielectric interface.

After modification, the as-grown 2D-BN was directly used to grow CVD-WSe_2_ for FET devices. The clean flat dielectric interface has low density of dangling bonds, charge impurities and charge traps, which lead to improved mobility compared with that of CVD-WSe_2_ grown on bare SiO_2_/Si. MD simulation shows that the 2D-BN can better bridge the vibrational spectrum across the dielectric interface, and protect the interfacial heat conduction against substrate roughness. Thus, the clean and conformal 2D-BN produced by ne-PECVD not only improves the device mobility but also reduces interfacial thermal resistance. After ne-PECVD modification, the interfacial thermal resistance of CVD-WSe_2_/SiO_2_ and CVD-MoSe_2_/SiO_2_ decreases by 4.55 × 10^−8^ and 1.21 × 10^−7^ m^2^ K W^−1^ to a value lower than 4.2 × 10^−8^ and 1.1 × 10^−7^ m^2^ K W^−1^, respectively. As a result, the saturated power intensity of the CVD-WSe_2_ FET increases by several folds up to 4.23 × 10^3^ W cm^−2^. More importantly, the dielectric interface with ne-PECVD 2D-BN exhibits lower interfacial thermal resistance than that with post-growth transferred CVD 2D-BN, indicating the great importance of the clean conformal interface in efficient thermal dissipation. Therefore, the ne-PECVD modification results in electrical devices with both high performance and power stability, compared with that on a bare SiO_2_ or CVD 2D-BN dielectric surface. These, as well as the advantages of low modification temperature, atomically smooth and clean surface, no requirement of post-growth transfer, conformal growth on 3D surface, capability for scaling up and industrial compatibility with microelectronic process, make this approach an ideal dielectric interface modification methodology for future micro/nanoelectronics.

## Methods

### Growth of 2D-BN

Continuous 2D-BN films were produced on SiO_2_/Si in a PECVD system. Ammonia borane (BH_3_-NH_3_, Sigma-Aldrich), which was used as the precursor, was placed in an isolated semi-enclosed quartz tube in *T*1 zone. Semi-enclosed quartz tube was used to reduce the precursor feeding rate and to obtain a steady precursor supply. A clean SiO_2_/Si was placed in *T*2 zone and heated to 500 °C in a constant Ar/H_2_ flow of 100 sccm/10 sccm (860 mTorr). After the *T*2 reached 500 °C, 20 mg ammonia borane was heated up to 110 °C. Meanwhile, the plasma generator (30 W) was opened, and the plasma flame filled the whole quartz tube, resulting in high efficient growth of 2D-BN. After growth for 30–60 min, the furnace was fast cooled to room temperature.

### Characterization

The 2D-BN and WSe_2_ samples were measured by AFM (Multimode 8, Bruker, tapping mode), high-resolution TEM (Tecnai G2 F20 S-Twin, acceleration voltage: 200 kV), EDS (equipped on TEM), optical microscopy (DM2500P, Leica), XPS (Perkin-Elmer PHI 5300 with 250 W Mg Kα source, 1253.6 eV), Raman spectra (HORIBA XploRA, 532 nm laser), UV-vis absorption spectrophotometer (Perkin-Elmer, Lambda35), and field-emission SEM (ZEISS, Ultra 55). The thermal images were measured by using a SThM probe (VITA-DM) mounted in the tip cantilever of Bruker Dimension Edge AFM (Supplementary Note 7, Supplementary Fig. 35). For TEM, AFM, or STM measurements, PMMA was spin-coated on the 2D-BN/SiO_2_/Si, and then the PMMA/2D-BN film was lift off from SiO_2_/Si by etching in 20% hydrofluoric acid. The PMMA/2D-BN film was rinsed in deionized water and transferred to the TEM grids, SiO_2_/Si, or HOPG, respectively. Finally, the PMMA was removed by either immersion in acetone or heating at 400 °C in Ar atmosphere. STM measurements were carried out in a custom-built multi-chamber ultrahigh vacuum system housing an Omicron LT-STM in the analysis chamber with a base pressure better than 1.0 × 10^−10^ mbar. All STM images were recorded in constant current mode at liquid nitrogen temperature (77 K) using electrochemically etched tungsten (W) tips. The STS data were acquired using a lock-in amplifier by applying a small sinusoidal modulation to the tip bias voltage (typically 3 V at 600 Hz). All STM images were processed using WS_x_M.

### Device fabrication and measurement

CVD-WSe_2_ was directly grown on 2D-BN/SiO_2_/Si by CVD. PT-WSe_2_ was prepared by post-growth transfer of CVD-WSe_2_ to 2D-BN/SiO_2_/Si using PMMA. After that, the source-drain electrodes (5/50 nm Cr/Au or 50 nm Au) were patterned on the sample by electron beam lithography and thermal deposition (Kurt J. Lesker). To obtain a better contact between the sample and the electrodes, thermal-annealing or current-annealing was performed. The electrical measurement was carried out in air at room temperature by a probe station (EVERBING, PE-4) and a semiconductor analyzer (Keysight B1500A). Details of the differential 3*ω* measurement were discussed in Supplementary Note 5.

## Supplementary information

Supplementary Information

## Data Availability

The data that support the findings of this study are available within the article and its Supplementary Information File or available from the corresponding author.
