# Supplementary material for: Conformal hexagonal-boron nitride dielectric interface for tungsten diselenide devices with improved mobility and thermal dissipation
Source: Nat Commun. 2019 Mar 13;10:1188. doi: 10.1038/s41467-019-09016-0 (PMC6416324; doi:10.1038/s41467-019-09016-0)
Supplement: Supplementary file 1 — Supplementary Information [file 41467_2019_9016_MOESM1_ESM.pdf]

Supplementary Information for

"Conformal Hexagonal-Boron Nitride Dielectric Interface for Tungsten Diselenide Devices with Improved Mobility and Thermal Dissipation"

Liu et al.

Supplementary Note 1. Detailed discussion of the near-equilibrium growth mechanism

Controlled experiments show that a low and steady precursor feeding rate is the pivotal factor for the growth of 2D-BN. As a result, solid ammonia borane is placed in a small quartz tube with one end sealed, and *T*1 remains below 115 °C. If *T*1 increases above 120 °C, a disordered *h*-BN film with a rough surface (Supplementary Fig. 19) is obtained on SiO_2/Si. The pink color, as well as an optical energy gap of 5.71 eV calculated according to the UV-vis spectroscopy (Supplementary Fig. 20) indicate that the sample is a thick *h*-BN film rather than 2D-BN, similar to the results in previous literatures[1-4]. This result is attributed to the metal catalyst-free growth mechanism of PECVD. In a metal-catalysed CVD, the transition metal catalyses the decomposition of precursor molecules and reduces the threshold barrier for 2D-BN crystal growth, thus efficient growth takes place on metals (growth temperature: 800~1000 ºC) rather than on inert surfaces (growth temperature: 1100~1400 ºC), energetically and kinetically[5,6]. In this work, ammonia borane is evaporated (H_2, monomeric aminoborane, borazine, etc. also exist owing to thermal decomposition)[7], and then decomposed into boron and nitrogen species (radicals, ions, atoms) by the plasma. These highly reactive species overcomes the large threshold barrier required, leading to metal catalyst-free growth of BN materials directly on the inert surface at low temperature. However, these species are inclined to form structural defects on edges, which prevent the growth of 2D-BN crystals. As demonstrated previously[8,9], there is a competition between the crystal growth and etching in the PECVD growth of graphene. The plasma removes the edge defects generated and keep the edges smooth and active in the whole growth process. Thus in a critical equilibrium state of the competition, efficient metal catalyst-free growth of graphene crystals is realized. In fact, some literatures have demonstrated the etching of BN by H_2, Ar, O_2 or H_2/Ar plasma[10-12], and we deduce a similar competition process exists in the growth of 2D-BN. Different from graphene, 2D-BN lattice is

energetically highly stable. Experiments show no obvious etching of 2D-BN in H_2/Ar plasma even after 60 min treatment (Supplementary Fig. 18). Thus, the nucleation/deposition effect dominates, and a critical equilibrium state similar to that of graphene growth is difficult to establish. In a non-equilibrium state, disordered or amorphous BN films are typically obtained as shown in Supplementary Fig. 19, 20, similar to the results reported previously[1-4]. Nevertheless, the literature and first-principle calculation (Supplementary Note 2) show that the edge defect has a higher energy[13], compared with pristine *h*-BN lattice and H-passivated edges. The energy increases by 7.458 and 9.526 eV, when a B_3N_2 or B_2N_3 pentagonal defect forms on H-passivated armchair edges, respectively. As a result, the etching tends to occur at the edge defects. Owing to the low etching rate, a low and steady precursor feeding rate is required to establish a reversible competition between the nucleation/growth effect and the etching effect. Therefore, in a near-equilibrium state, moderate etching by the H_2/Ar plasma removes defects generated on the edges and keeps the edges atomically smooth and active during the whole PECVD process, resulting in efficient crystal growth of 2D-BN directly on inert surface without catalyst.

Raman measurement supports the near-equilibrium growth mechanism. The FWHM of the E^{2g} mode is related to the crystallinity of the 2D-BN, and the broadening of the peak is typical for small *h*-BN crystallites[14,15]. We produced 2D-BN by PECVD at different Ar/H_2 ratios, pressures, plasma powers and precursor feeding rates. The FWHM of the E^{2g} mode (Supplementary Fig. 22) shows weak dependence on the Ar/H_2 ratio, pressure and plasma power, and strong dependence on the precursor feeding rate. With lower precursor feeding rate ($T1$ decreases from 125 °C to 105 °C), the FWHM decreases from ~93 cm^{-1} to ~35 cm^{-1}. This result shows that low precursor feeding rate is pivotal to establish the near-equilibrium state between plasma etching and nucleation/growth for the growth of 2D-BN, owing to the weak etching effect. When the $T1$ is 105 °C, the FWHM reaches as low as 28 cm^{-1} (Supplementary Fig. 23), close

to that (~24 cm^{-1}) of CVD 2D-BN, showing the importance of the low precursor feeding rate in producing high quality 2D-BN.

Supplementary Note 2. First-principles calculation of the 2D-BN with different edge structures

The first-principles calculations were based on the density functional theory (DFT) using the Perdew-Burke-Ernzerhof (PBE)[16] realization of the generalized gradient approximation (GGA) for the exchange-correlation, as implemented in the Vienna Ab-initio Simulation Package (VASP)[17]. The projector augmented wave (PAW) method was employed to model the ionic potentials[18]. Kinetic energy cutoff was set above 500 eV for all calculations. A vacuum space above 25 Å was introduced to avoid interactions between images. The structure was fully optimized with respect to the ionic positions until the forces on all atoms were less than 0.01 eV/Å. The Monkhorst−Pack k-point sampling was used for the Brillouin zone integration. The calculated models are shown in Supplementary Fig. 21.

Supplementary Note 3. The calculation of the FET mobility.

The mobility was calculated with the equation below at the linear regime:

$$\mu = \left(\frac{L}{WC_i V_{ds}}\right)\left(\frac{\Delta I_{ds}}{\Delta V_g}\right) \qquad \text{(S1)}$$

where C_i is the dielectric capacitance, W is channel width, and L is channel length.

For instance, Supplementary Fig. 27 shows a CVD-WSe_2 FET devices on 2D-BN/SiO_2/Si substrate. L is 7.9 μm, W is 25.5 μm, C_i is 9.98×10^{-9} F cm^{-2}, V_{ds} is −2 V, $\Delta I_{ds}/\Delta V_g$ is 7.8×10^{-6} A V^{-1}. The calculated mobility is around 121 cm^2 V^{-1} s^{-1}.

It is worth noting that the 2D-BN/SiO_2/Si has two dielectric layers. The thickness of SiO_2 used in this work is 300nm, while the thickness of the monolayer 2D-BN is about

0.85nm. The C_i of 300 nm SiO_2 is about 10 nF cm^{-2}. The capacitance of monolayer 2D-BN was calculated by:

$C_{2D\text{-}BN} = k\ \varepsilon_0 / d$ (S2)

where k is the dielectric constant of h-BN (the value is about 4.0)[19], ε_0 is the permittivity, and d is the thickness of 2D-BN (about 0.85nm according to the AFM image). As a result, the C_i of monolayer 2D-BN was 4164 nF cm^{-2}.

Therefore, the two layer system in series contributes to the total capacitance (C_{total}) of 9.98 nF cm^{-2} based on the equation:

$1 / C_{total} = 1 / C_{SiO2} + 1 / C_{2D\text{-}BN}$ (S3)

indicating the contribution of 2D-BN on the capacitance is negligible[20].

Supplementary Note 4. Discussion of the SThM results.

Experimental details of the SThM measurement are shown in Supplementary Note 7. An active and contact mode is used in the measurement while temperature changes ΔT in the tip is monitored when scanning on the sample surface. To obtain a better thermal image, the ΔT on 2D-BN and SiO_2/Si substrate is set to zero by adjusting the $R3$ in the Wheatstone bridge (see details in Supplementary Note 7). The Joule heat, generated at the tip, dissipates via two directions: tip to cantilever and tip to sample. The former can be assumed to be the same if the heating power is a constant. The latter will path through sample ($MoSe_2$ or WSe_2) to interface and finally dissipate into the substrate, suggesting that larger interfacial thermal resistance between sample and substrate will result in larger value of ΔT. As shown in the 2D-mapping thermal image of CVD-WSe_2/2D-BN/SiO_2, CVD-WSe_2/SiO_2, CVD-$MoSe_2$/2D-BN/SiO_2 and CVD-$MoSe_2$/SiO_2 (Fig. 6a-6d), a higher value of ΔT is observed for CVD-WSe_2/SiO_2 or CVD-$MoSe_2$/SiO_2 compared with that of CVD-WSe_2/2D-BN/SiO_2 and CVD-$MoSe_2$/2D-BN/SiO_2 (Fig. 5e). To minimize the measurement uncertainty and verify the

data reproducibility, we measured 47, 50, 60 and 40 samples or locations of CVD-WSe_2/2D-BN/SiO_2, CVD-WSe_2/SiO_2, CVD-$MoSe_2$/2D-BN/SiO_2 and CVD-$MoSe_2$/SiO_2, respectively. The measurement statistics of the ΔT change (temperature difference between sample and substrate) is presented in Fig. 5f, and the data scatter is mainly attributed to measurement uncertainty of ΔT and roughness differences on sample surfaces. No obvious ΔT change is observed on 2D-BN/SiO_2, owing to efficient thermal dissipation between the tip and the substrate across the 2D-BN. Samples ($MoSe_2$ or WSe_2) on 2D-BN/SiO_2/Si have much lower temperature rise than that on SiO_2/Si, indicating that the 2D-BN layer helps thermal dissipation from tip to substrate across the sample/dielectric interface.

Supplementary Note 5. Experimental details of differential 3ω measurement.

A 3μm-wide Cr/Au (5nm/50nm) electrode was deposit onto $MoSe_2$ or WSe_2 through electron beam lithography and thermal evaporation process (Fig. 7a, Supplementary Fig. 32). Next, high dose O_2 plasma was used to oxide $MoSe_2$ or WSe_2 layer and remove 2D-BN layer, to make sure that heat dissipates only in vertical direction. This process is crucial for 3ω measurement where one should assume heat flow only in one direction.

To carry out the 3ω measurement, an Ac-current with a frequency of ω was applied into the electrode. The Joule heat power can be treated to have a fluctuation with a frequency of 2ω, introducing a temperature fluctuation with a frequency of 2ω, i.e. $T_{2\omega}$. The resistance of Cr/Au electrode can be regarded to have a linear dependence with temperature. As a result, an AC voltage with a frequency of 3ω can be detected, which is related to the interface thermal resistance underneath the electrode and the thermal resistance of the substrate. The temperature increase of the electrode $T_{2\omega}$ can be calculated from:

$$T_{2\omega} = 2\frac{\mathrm{d}T}{\mathrm{d}R}\frac{R}{V}V_{3\omega} \qquad \text{(S4)}$$

where R is the resistance of the electrode, T is the temperature, V and $V_{3\omega}$ are the measured voltage with frequency of 1ω and 3ω, respectively.

The calculated thermal resistance (4.2×10^{-8} m^2 K W^{-1} in Fig. 7c) is the sum of the substrate thermal resistance and the interfacial thermal resistance of CVD-WSe_2/2D-BN/SiO_2. Therefore, the actual interfacial thermal resistance of CVD-WSe_2/2D-BN/SiO_2 is lower than 4.2×10^{-8} m^2 K W^{-1}. It is difficult to measure the interfacial thermal resistance of CVD-WSe_2/SiO_2 interface and CVD-WSe_2/2D-BN/SiO_2 interface directly. However, the difference of the interfacial thermal resistance of CVD-WSe_2/SiO_2 interface and CVD-WSe_2/2D-BN/SiO_2 interface can be detected by differential 3ω method. To carry out the differential 3ω method, the electrodes were fabricated both on CVD-WSe_2/SiO_2 interface and CVD-WSe_2/2D-BN/SiO_2 interface. The differential interfacial thermal resistance can be calculated from:

$$R_{\mathrm{int}} = \frac{\Delta T_{2\omega}\cdot S}{P} \qquad \text{(S5)}$$

where R_{int} is differential interfacial thermal resistance between CVD-WSe_2/SiO_2 interface and CVD-WSe_2/2D-BN/SiO_2 interface, S is cross section area between electrode and WSe_2, P is the Joule heat power and $\Delta T_{2\omega}$ is the $T_{2\omega}$ difference between CVD-WSe_2/SiO_2 interface and CVD-WSe_2/2D-BN/SiO_2 interface.

Supplementary Note 6. MD simulation of the interfacial thermal dissipation.

All MD simulations in this work are performed by using LAMMPS package[21]. A Tersoff potential is used to model Si-O bond in the substrate[22], and another optimized Tersoff potential is used to model B-N bond in hexagonal BN layer[23]. The internal interactions between WSe_2 layer are described by Lennard-Jones (LJ) potential

$V(r_{ij}) = 4\varepsilon[(\sigma_{ij}/r_{ij})^{12} - (\sigma_{ij}/r_{ij})^{6}]$, the parameters is from the work by Shen *et al*[24]. The interlayer non-bonded interactions are also described by LJ potential, which are obtained from the Universal Force Field (UFF)[25]. The cut-off distance in the LJ potential is set as $2.5\sigma_{ij}$ for all kinds of bonds. In the MD simulations, the size of amorphous SiO_2 substrate is 12.4 nm × 12.4 nm × 4 nm, meanwhile the single-layer 2D-BN and WSe_2 are well covered the substrate, as shown in the Supplementary Fig. 34. Moreover, in order to simulate the practical surface roughness of SiO_2 substrate, we randomly moved surface atoms in a degree of *R*a.

In this work, we have used a transient thermo-reflectance (TTR) method[26] to calculate the interfacial thermal resistance, which has been previously applied to study the interfacial thermal transport between two-dimensional materials and their substrates[27,28]. After the hybrid WSe_2/SiO_2 and WSe_2/2D-BN/SiO_2 structure is fully relaxed at temperature 300 K, a thermal impulse is imposed on the supported WSe_2 layer to 390 K. In the following 300 ps thermal relaxation, the energy decay profile of WSe_2 and temperature variation of SiO_2 substrate and WSe_2 are recorded, and then fitted by using the following integral form[27,28]:

$$E_t = E_0 + (A/R) \cdot \int_0^t \left(T_{WSe_2} - T_{SiO_2}\right) dt \qquad \text{(S6)}$$

where E_0 is the initial energy of the fitting process and A is the contact area; The total energy (E_t), temperature T_{WSe2} and T_{SiO2} from MD simulations are illustrated in Supplementary Fig. 35. It can be observed that the fitting curve soundly matches the calculated MD results, indicating the validity of eq. (S6) to describe this transport process.

Supplementary Note 7. Experimental details of the SThM measurement.

We obtained the thermal image by using a SThM probe (VITA-DM) mounted in the tip cantilever of Bruker Dimension Edge AFM. The SThM probe was specially designed as contact mode probe that incorporate a metal resistive film near the apex of the probe, with the temperature coefficient of 0.196 ± 0.005 Ω/K above room temperature. The SThM measurement worked at the contact mode with 5 Hz scan rate. A large steady electrical current was applied to the tip, which realized heating and measuring the resistance at the same time. A schematic of SThM is shown in Supplementary Fig. 36. A constant output voltage of 1.5V was applied on to the Wheatstone bridge. The measured voltage of SThM tip, ΔV, could be adjusted by changing the value of $R3$. To obtain a better 2D-mapping thermal image, the ΔV (corresponding to the ΔT) was set to be near zero when the tip contacted with the SiO_2/Si or the 2D-BN surface.

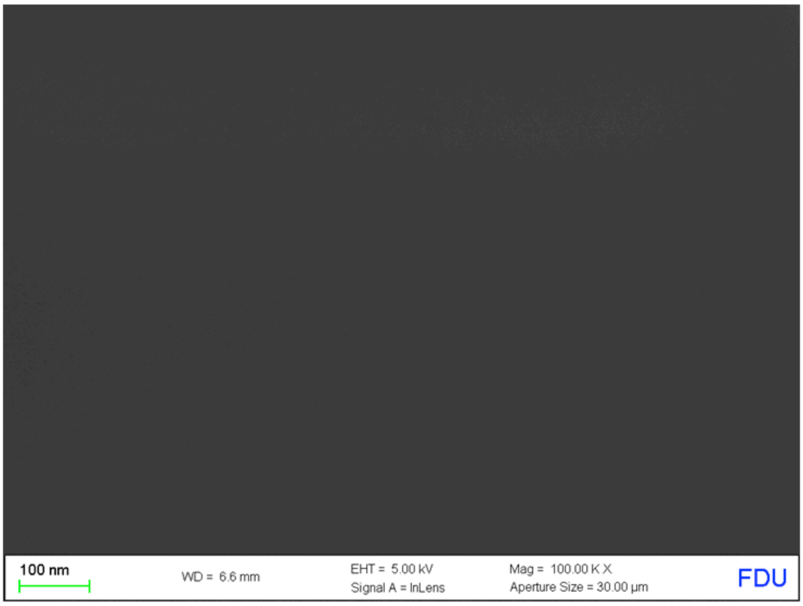

Supplementary Fig. 1. SEM image of a 2D-BN film grown on SiO_2/Si by ne-PECVD (30 min).

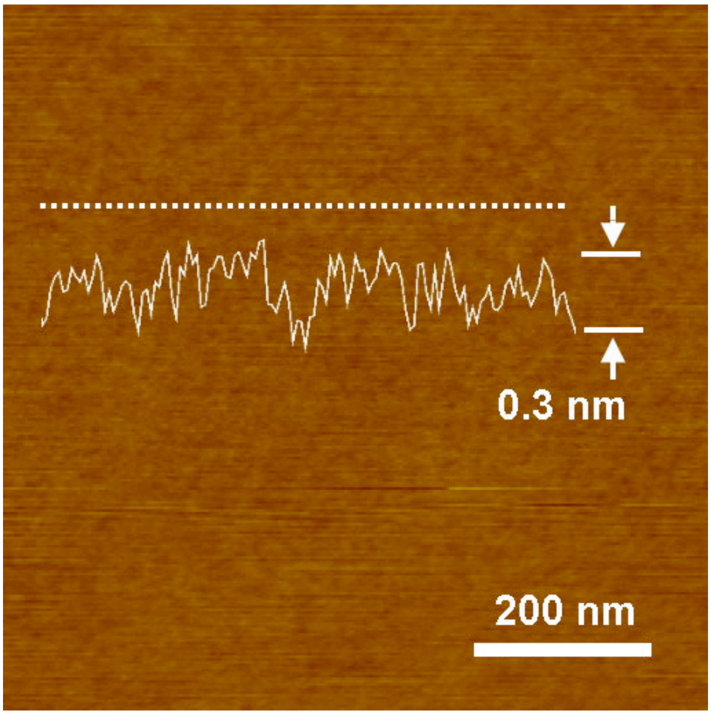

Supplementary Fig. 2. AFM image of a bare SiO_2/Si substrate. The scale bar is 200 nm.

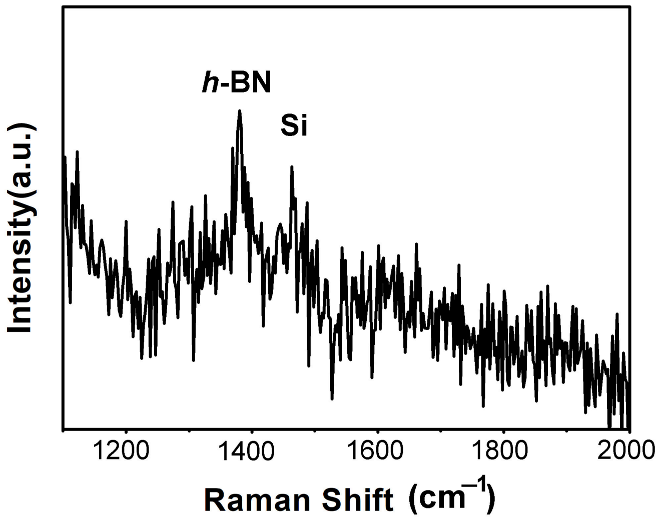

Supplementary Fig. 3. Raman spectrum of a 2D-BN film (produced by CVD on Cu) transferred to SiO_2/Si.

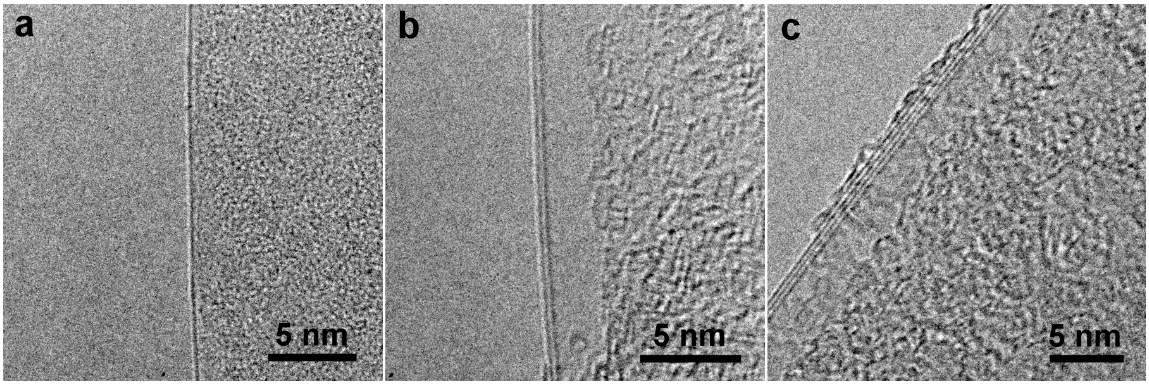

Supplementary Fig. 4. High resolution TEM images of 2D-BN membranes produced by ne-PECVD. **a,** 2D-BN grown on SiO_2/Si at 500 °C for 30 min. **b,** 2D-BN grown on SiO_2/Si at 500 °C for 40 min. **c,** 2D-BN grown on SiO_2/Si at 500 °C for 50 min. The scale bars are 5 nm.

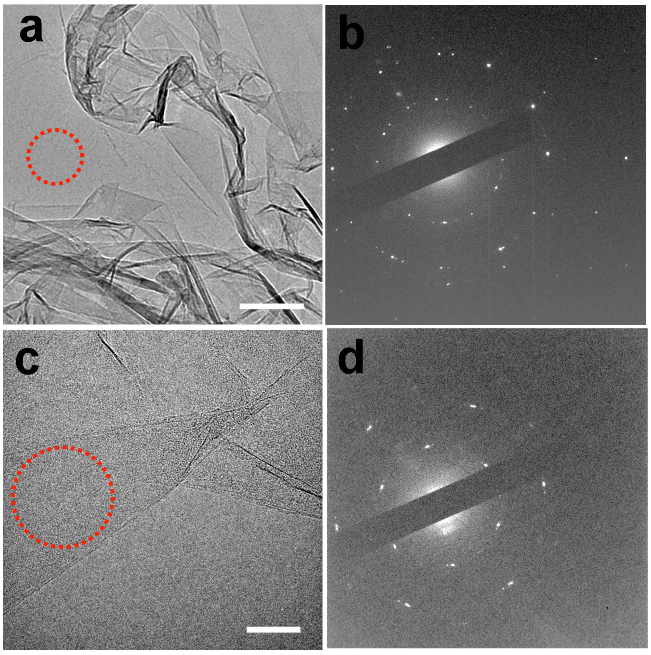

Supplementary Fig. 5. TEM images and selected area electron diffraction patterns of 2D-BN film grown by ne-PECVD. The selected area electron diffraction patterns in (b) and (d) are collected from the regions marked by dashed circles in (a) and (c), respectively. The scale bars are 200 nm in (a), and 20 nm in (c).

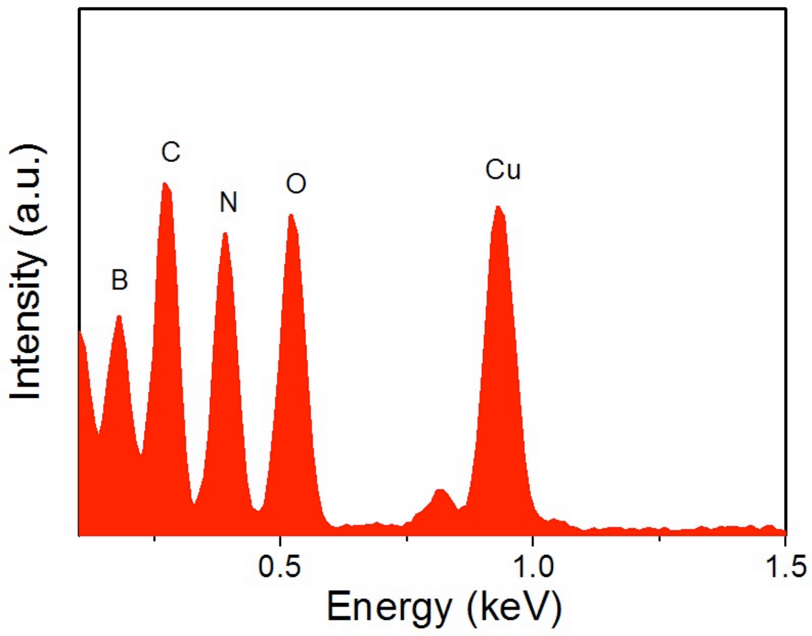

Supplementary Fig. 6. EDS spectrum of a 2D-BN film transferred to a carbon-copper TEM grid.

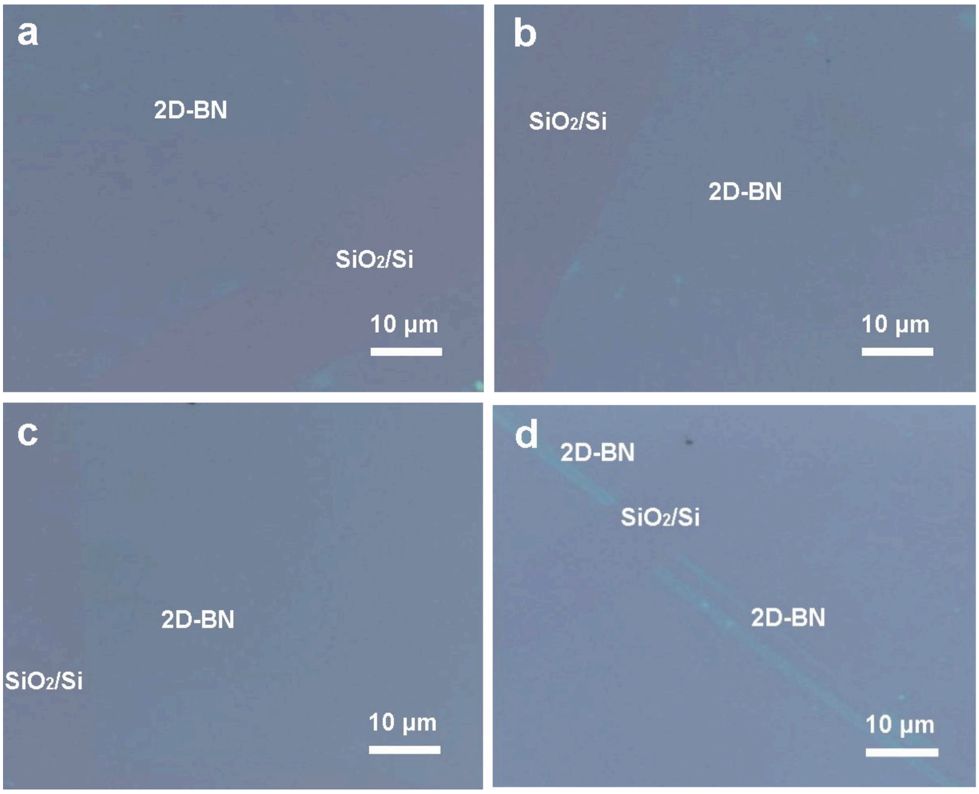

Supplementary Fig. 7. Optical image of 2D-BN films. **a-d,** The Optical images of 2D-BN films transferred to other SiO_2/Si substrates. The growth time is 30, 40, 50, 60 min, respectively. The scale bars are 10 μm.

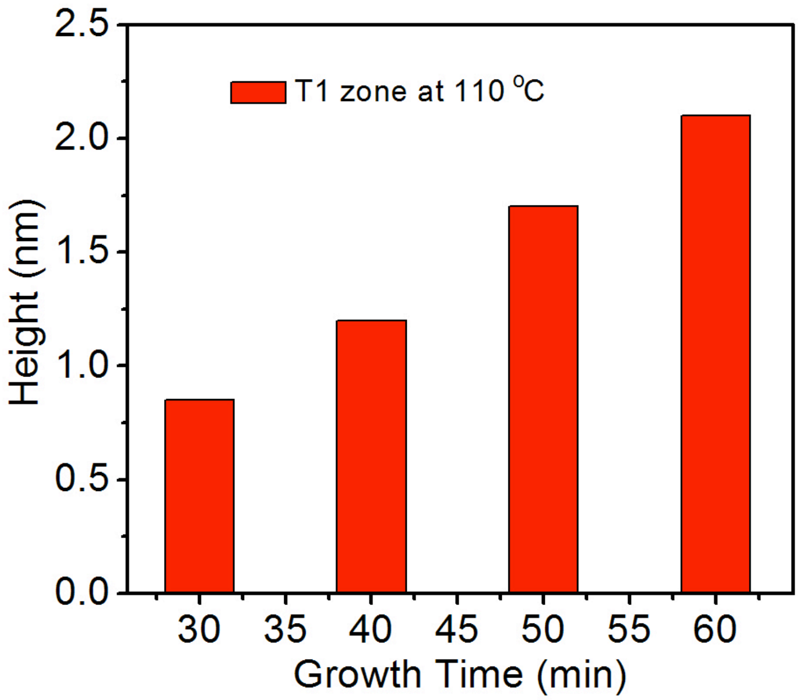

Supplementary Fig. 8. Relationship between the growth time and the thickness of 2D-BN.

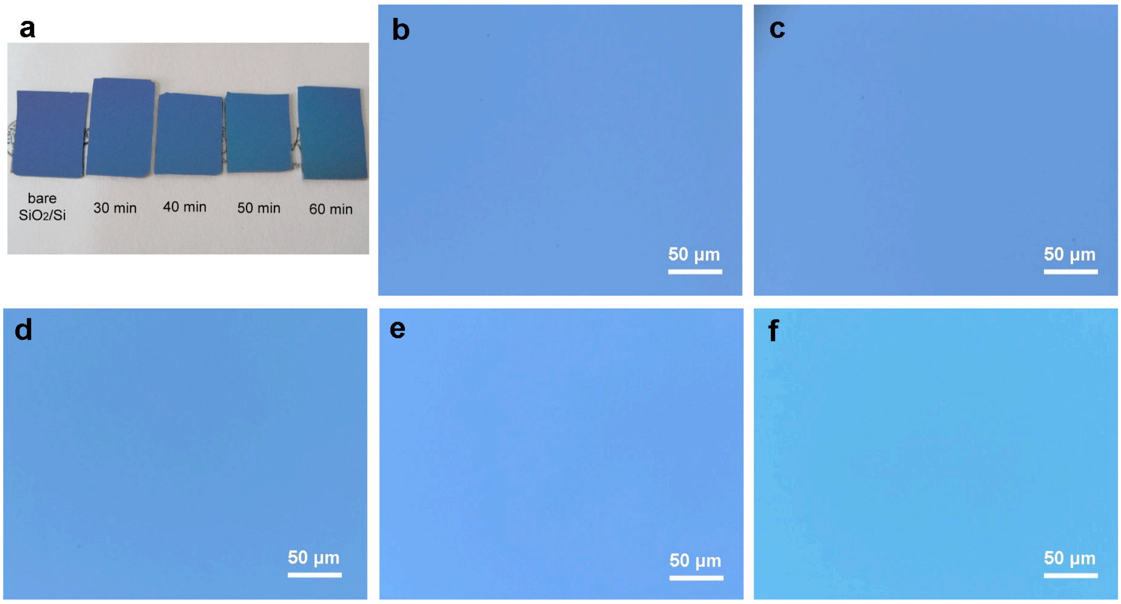

Supplementary Fig. 9. 2D-BN samples produced by us. **a,** Optical image of bare SiO_2/Si and 2D-BN films grown SiO_2/Si by ne-PECVD. **b-f,** Optical images of SiO_2/Si after 0, 30, 40, 50, 60 min growth, respectively. The scale bars are 50 μm.

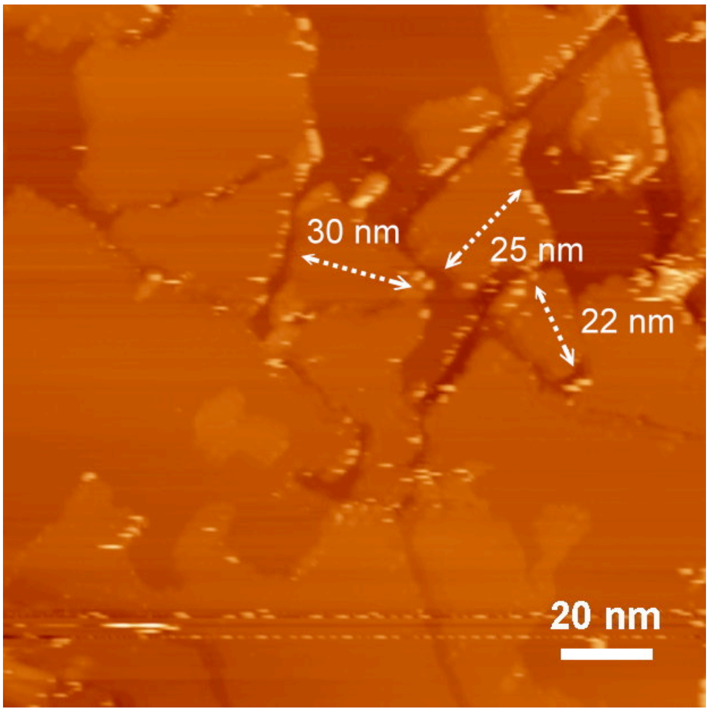

Supplementary Fig. 10. STM image of a 2D-BN film transferred to HOPG substrate. The tip bias is −2 V. The scale bar is 20 nm.

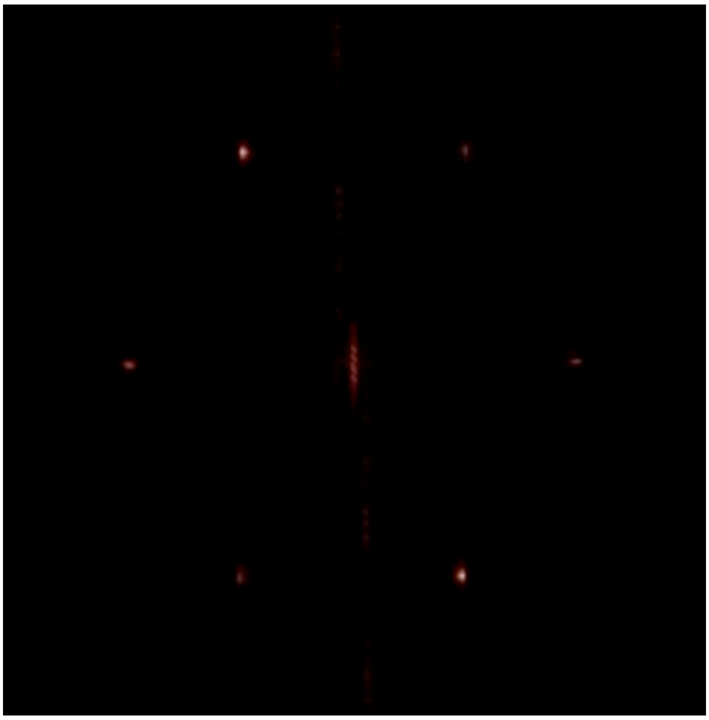

Supplementary Fig. 11. FFT pattern of the STM image (Fig. 2a).

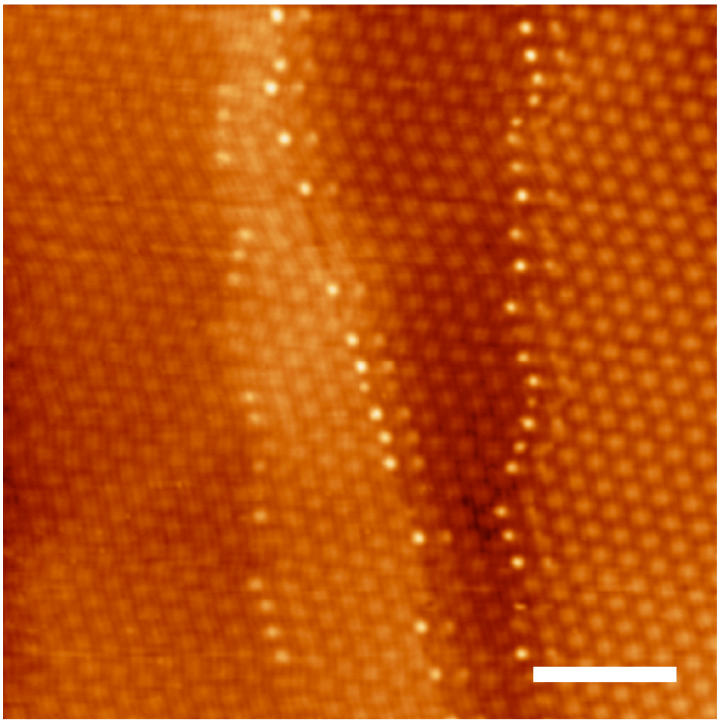

Supplementary Fig. 12. STM image of a 2D-BN film on HOPG. After transferring the 2D-BN to HOPG, Moiré pattern can be clearly observed, indicating highly crystalline nature of the 2D-BN domains. The scale bar is 20 nm.

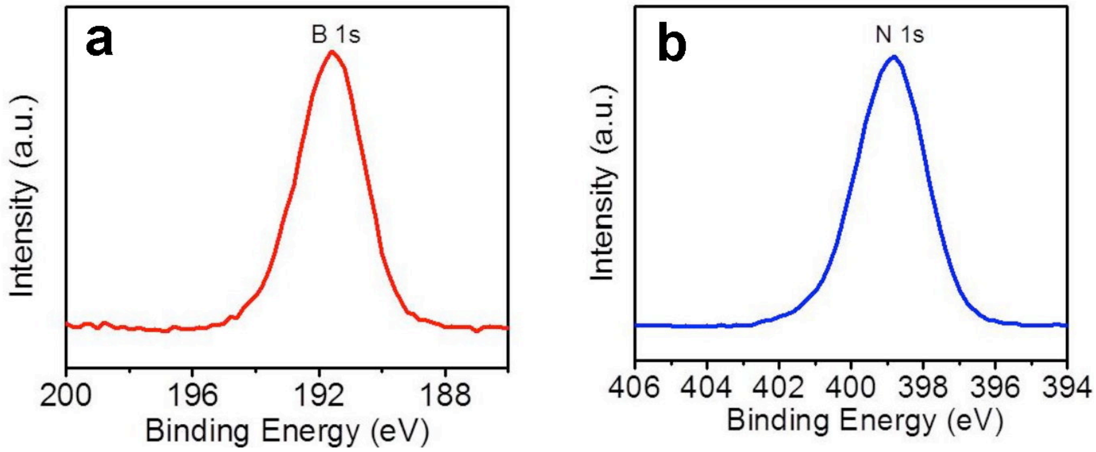

Supplementary Fig. 13. XPS spectra of the 2D-BN sample shown in **Fig. 2h**. **a,** XPS B1s and **b,** XPS N1s spectra of the 2D-BN sample.

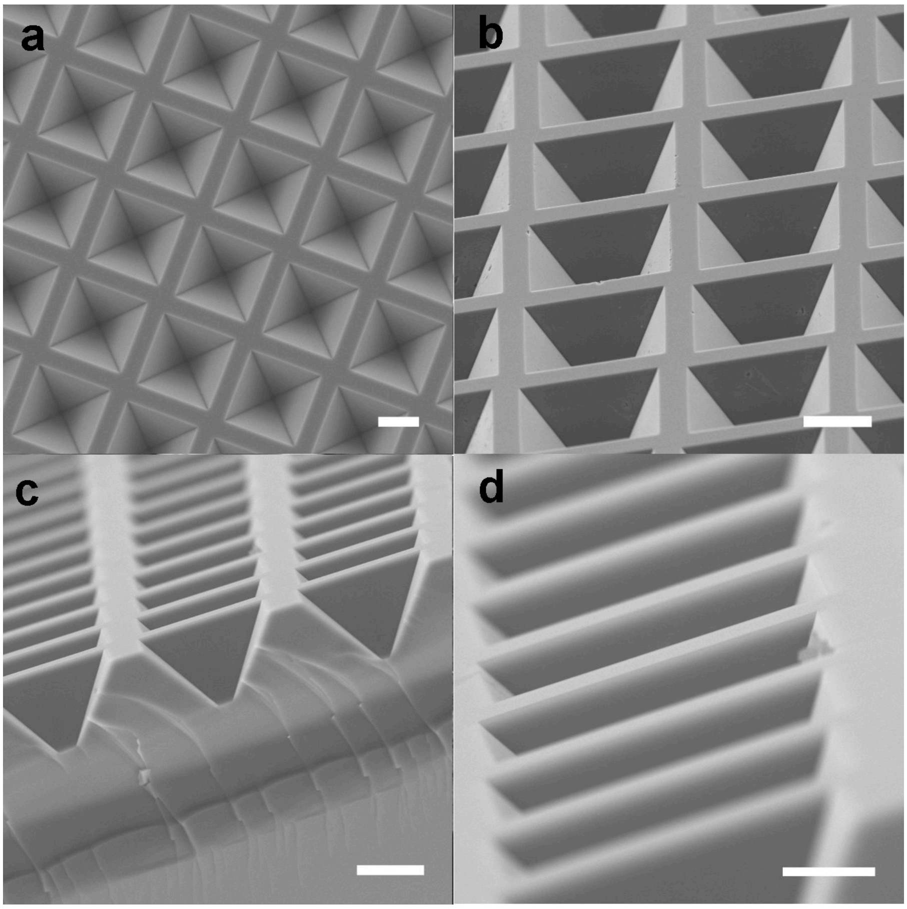

Supplementary Fig. 14. a, Top view and **b-d,** side view of SEM images of a 2D-BN film grown on SiO_2/Si with 3D pattern by ne-PECVD. No interstices, wrinkles or incompact contacts are observed. The scale bars are 2 μm in **a-c**, 1 μm in **d**.

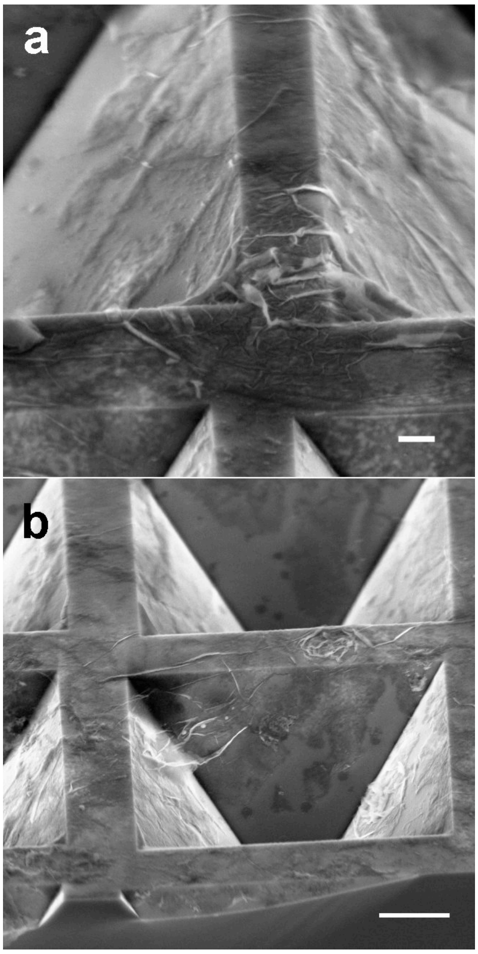

Supplementary Fig. 15. SEM images (side view) of a post-growth transferred 2D-BN film. After growth, the 2D-BN film was transferred from the growth substrate to the SiO_2/Si with 3D pattern. From the images, interstices, wrinkles and incompact contacts are observed. The scale bars are 200 nm in **a**, 1 μm in **b**.

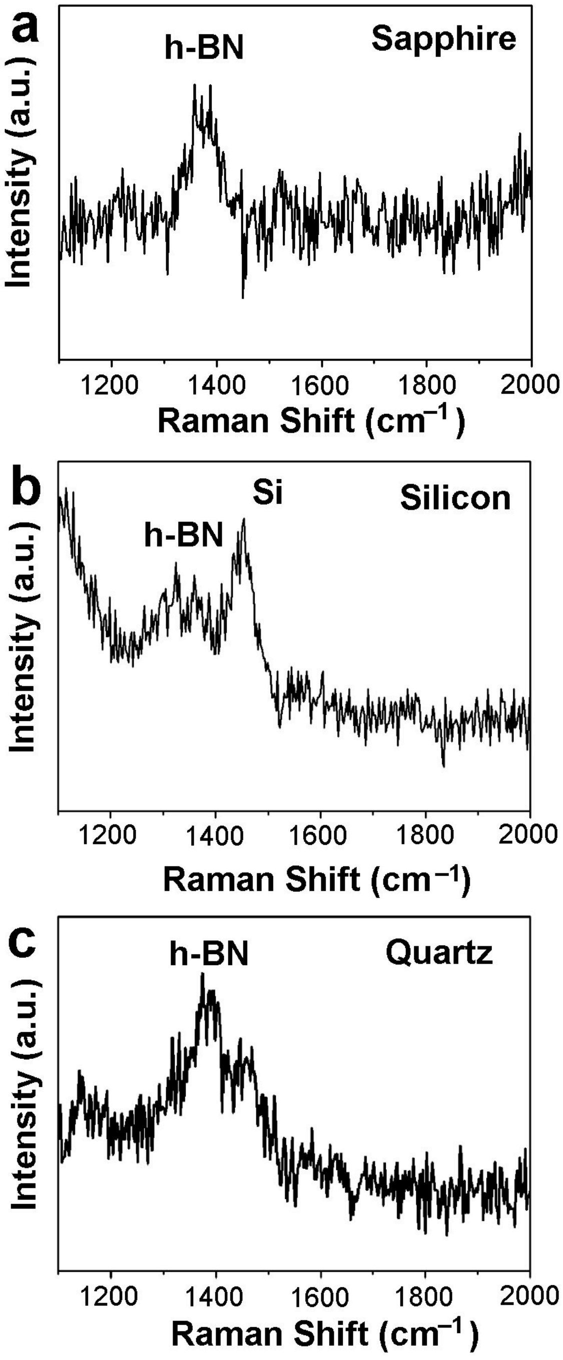

Supplementary Fig. 16. Raman spectra of 2D-BN grown on **a,** sapphire, **b,** silicon and **c**, quartz by ne-PECVD.

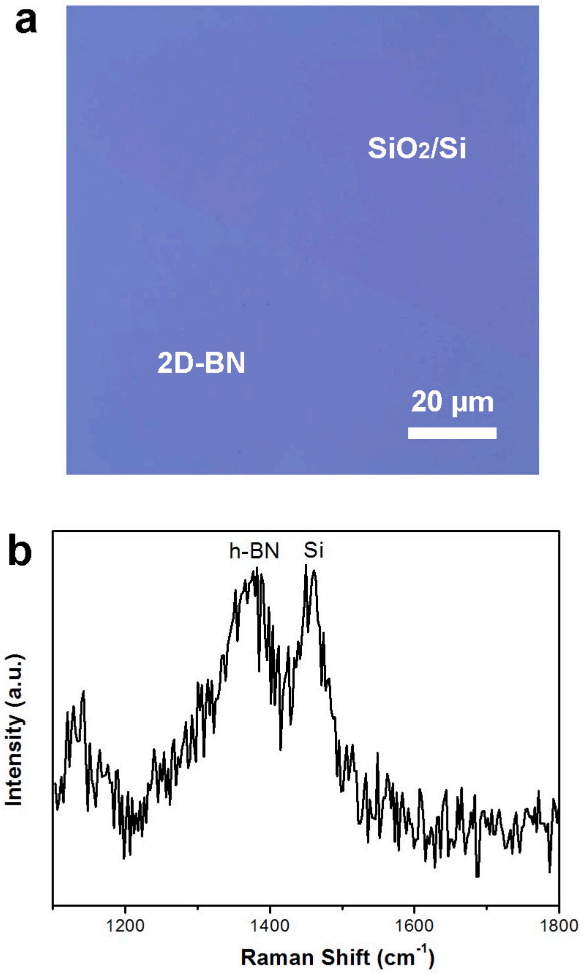

Supplementary Fig. 17. a, Optical image and **b,** Raman spectrum of a 2D-BN film produced by ne-PECVD at 300 °C. The 2D-BN film has been transferred to other SiO_2/Si substrate by PMMA. The scale bar is 20 μm.

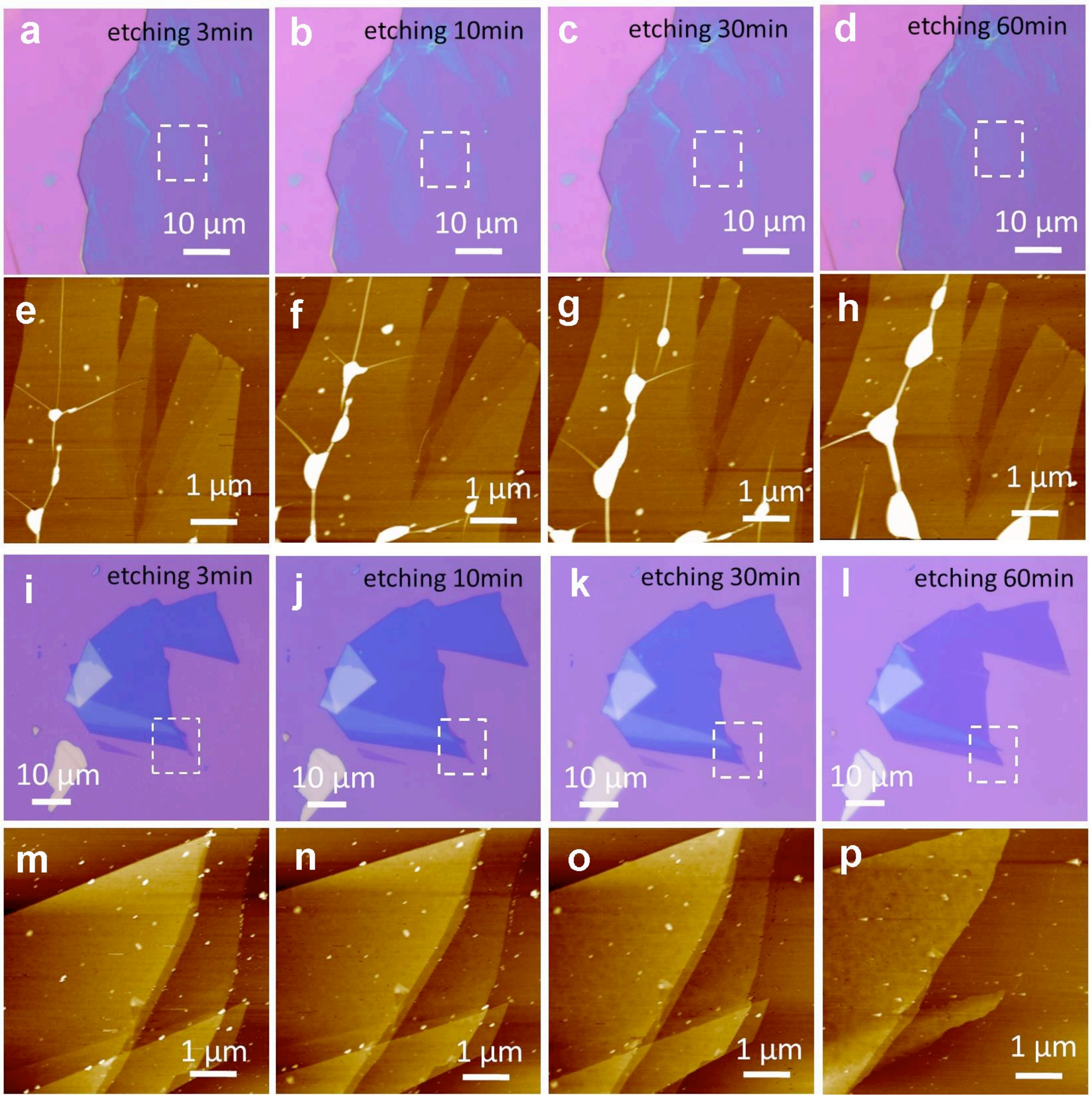

Supplementary Fig. 18. a-d, Optical images and **e-h,** AFM images of exfoliated 2D-BN after etching in Ar/H_2 plasma for 3, 10, 30, and 60 min, respectively. **i-l,** Optical images and **m-p,** AFM images of exfoliated graphene after etching in Ar/H_2 plasma for 3, 10, 30, and 60 min, respectively. The scale bars in **a-d**, **i-l** are 10 μm. The scale bars in **e-h**, **m-p** are 1 μm.

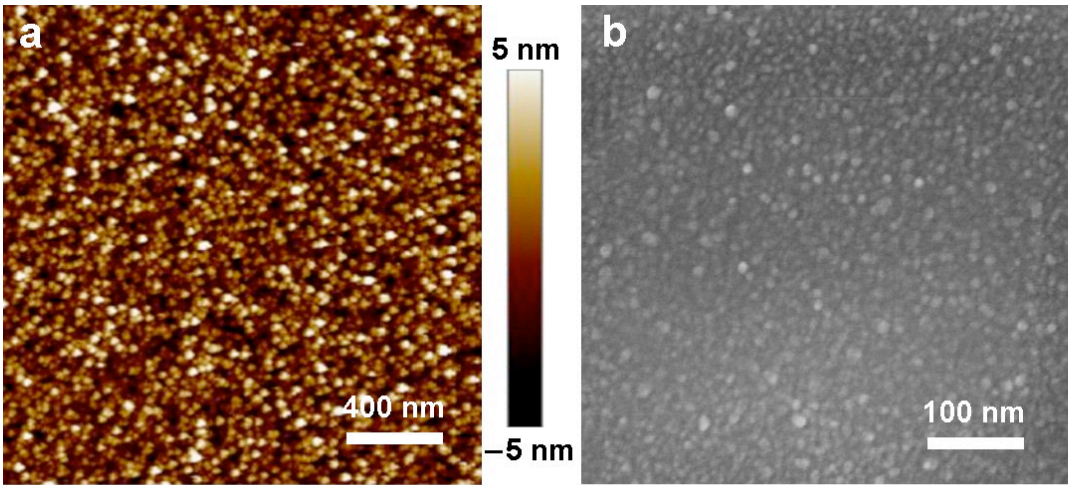

Supplementary Fig. 19. a, AFM and **b,** SEM image of a thick disordered *h*-BN film grown on SiO_2/Si by PECVD, when the temperature of *T*1 is 120 °C. The scale bars are 400 nm in **a** and 100 nm in **b**.

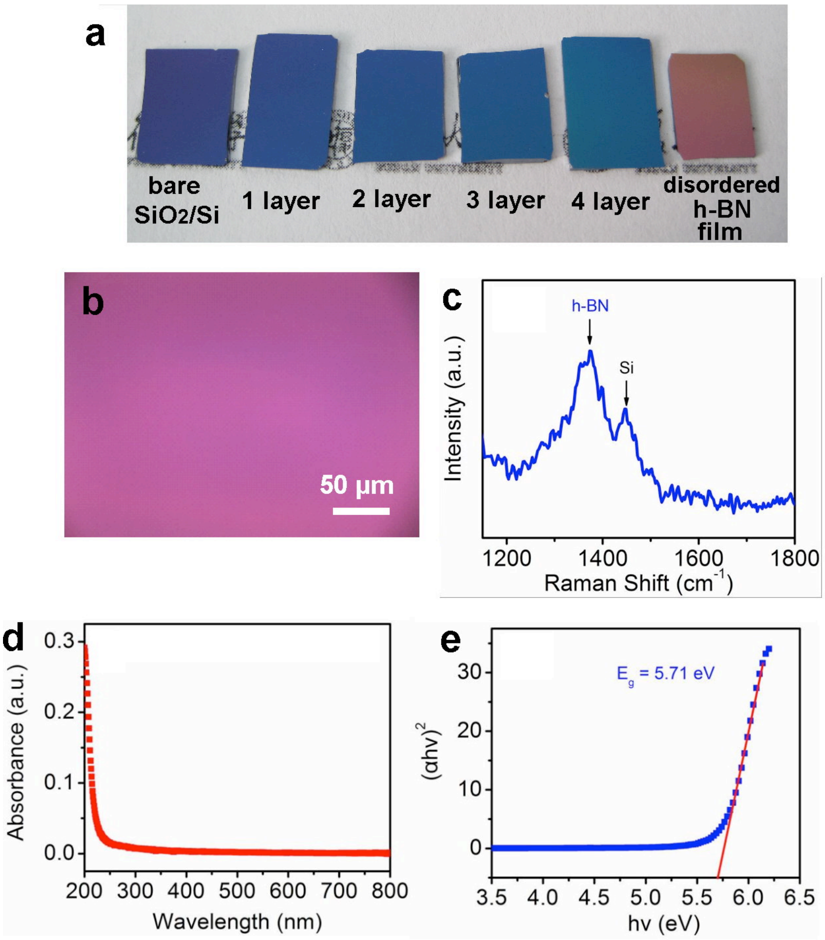

Supplementary Fig. 20. Thick h-BN samples produced by us. **a,** Optical image of *h*-BN films grown on SiO_2/Si. The right sample is prepared by PECVD (*T*1: 120 °C). **b,** Optical microscope image, **c,** Raman spectrum, **d,** room temperature UV−vis absorbance spectrum and **e,** Tauc plot of a thick disordered *h*-BN sample grown on SiO_2/Si by PECVD, when the temperature of T1 is 120 °C. The scale in **b** is 50 μm.

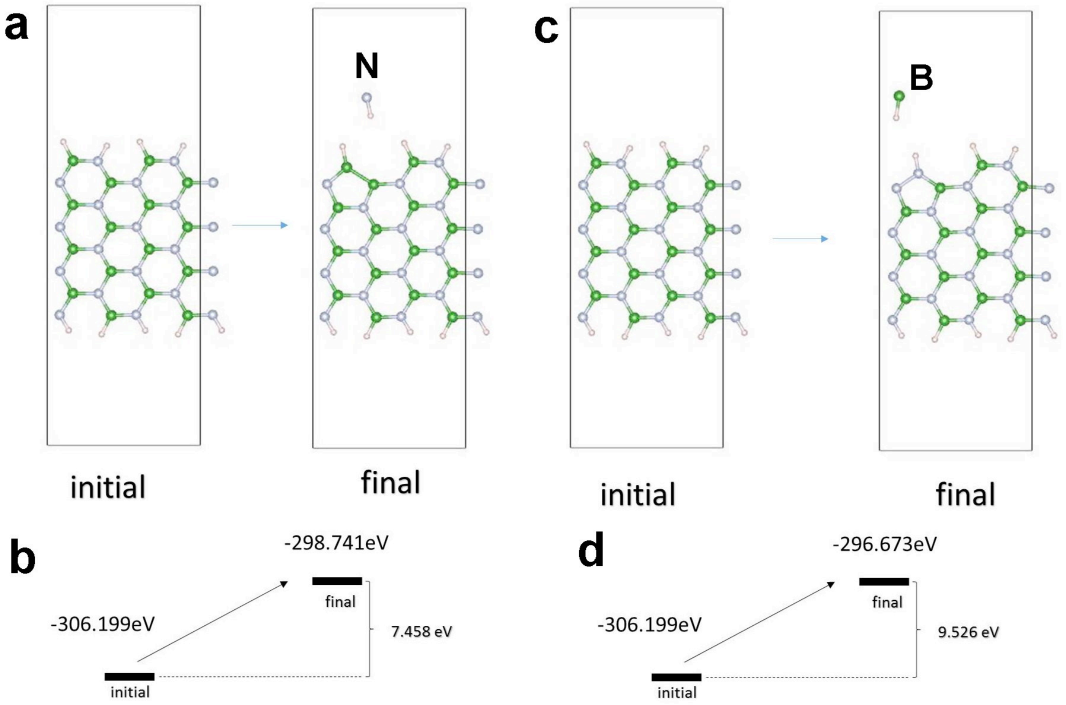

Supplementary Fig. 21. a, The atomic models and **b,** the calculated energies of a *h*-BN with hydrogen-passivated arm-chair edge and a *h*-BN with a B_3N_2 edge defect. **c,** The atomic models and **d,** the calculated energies of a *h*-BN with hydrogen-passivated arm-chair edge and a h-BN with a B_2N_3 edge defect.

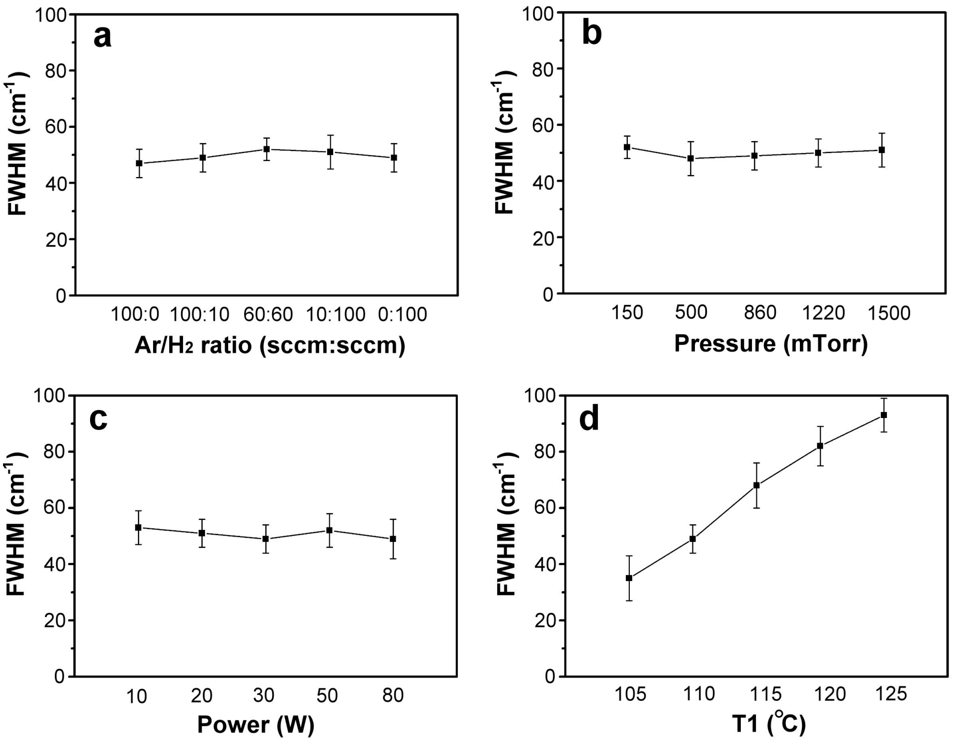

Supplementary Fig. 22. FWHM of the E^{2g} mode for the sample produced at different Ar/H_2 ratios, pressures, plasma powers and precursor feeding rates. (a) The samples are produced by PECVD (plasma power 30 W, 0.8±0.1 Torr, *T*1 = 110 ºC, growth temperature 500 ºC) at different Ar/H_2 ratio. (b) The samples are produced by PECVD (plasma power 30 W, Ar/H_2 ratio = 10:1, *T*1 = 110 ºC, growth temperature 500 ºC) at different pressure. (c) The samples are produced by PECVD (Ar/H_2 ratio = 10:1, 0.8±0.1 Torr, *T*1 = 110 ºC, growth temperature 500 ºC) at different plasma power. (d) The samples are produced by PECVD (plasma power 30 W, Ar/H_2 ratio = 10:1, 0.8±0.1 Torr, growth temperature 500 ºC) at different precursor feeding rates (*T*1 from 105 ºC to 125 ºC).

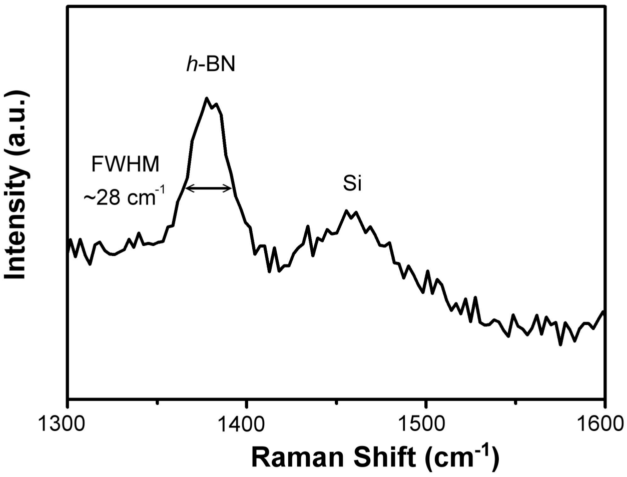

Supplementary Fig. 23. Raman spectrum of a sample produced by PECVD at low precursor feeding rates (*T*1 = 105 ºC).

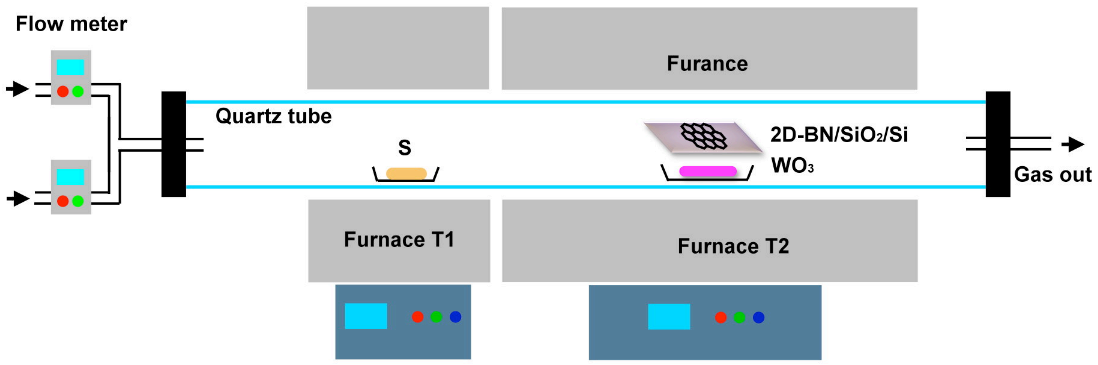

Supplementary Fig. 24. The illustration of the CVD system for growth of WSe_2.

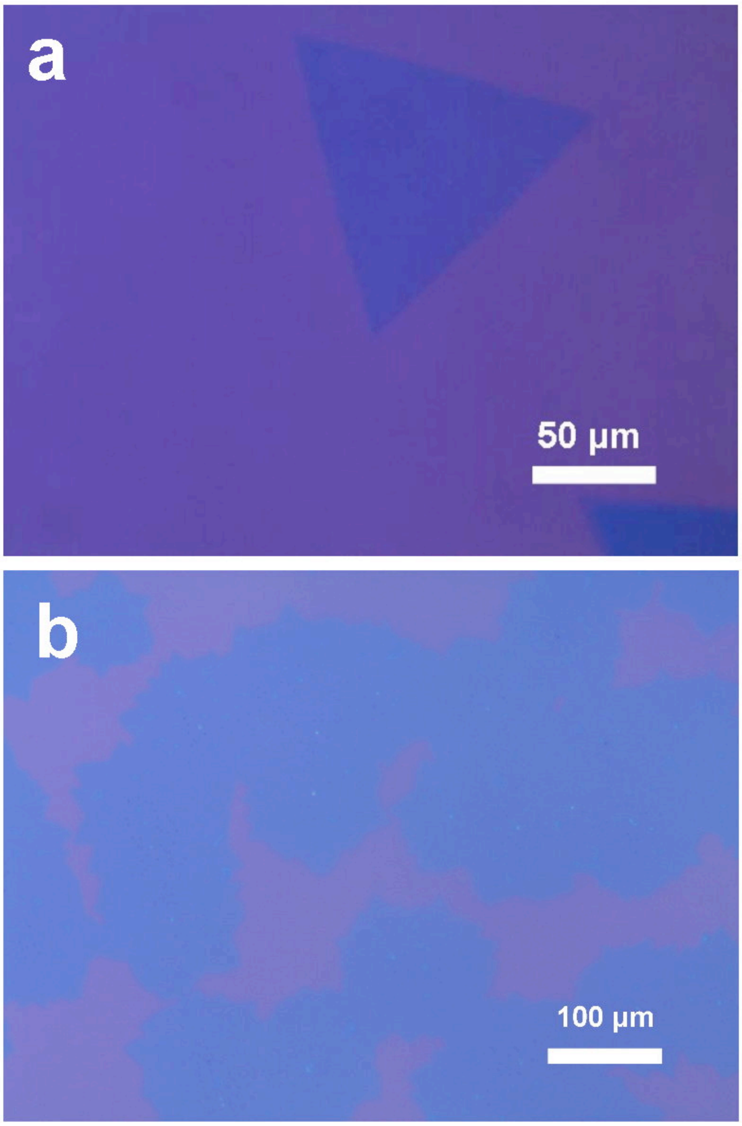

Supplementary Fig. 25. Optical images of CVD-WSe_2 crystals grown on 2D-BN/SiO_2/Si. The scale bars are 50 µm in **a**, 100 µm in **b**.

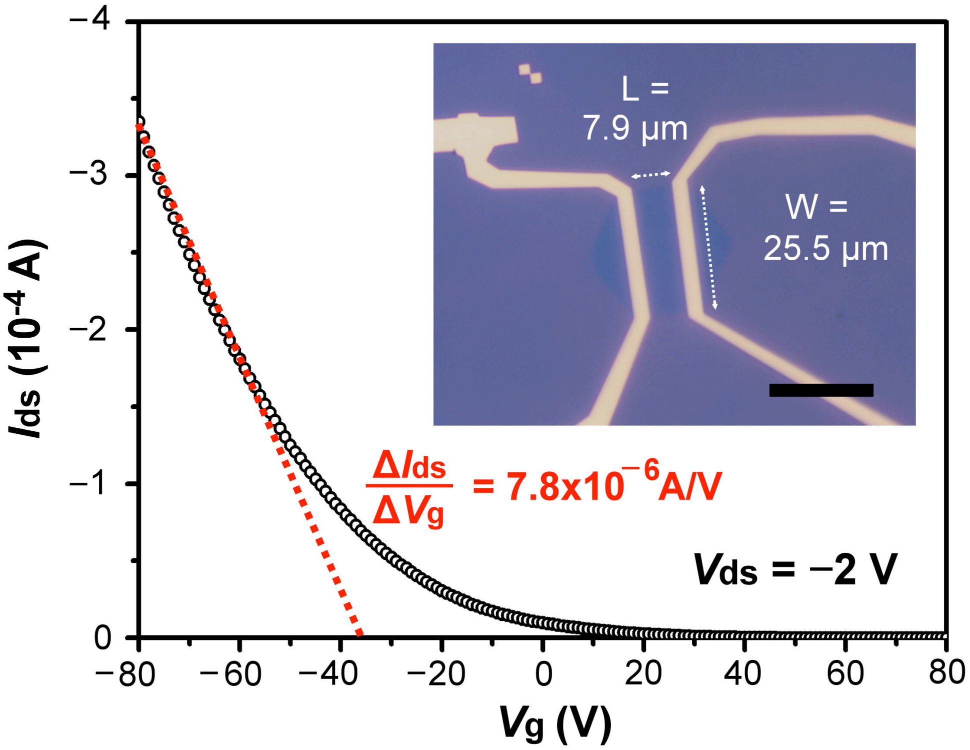

Supplementary Fig. 26. An optical image and the transfer curve of a CVD-WSe_2 FET devices using 2D-BN/SiO_2/Si substrate. The scale bar is 20 μm.

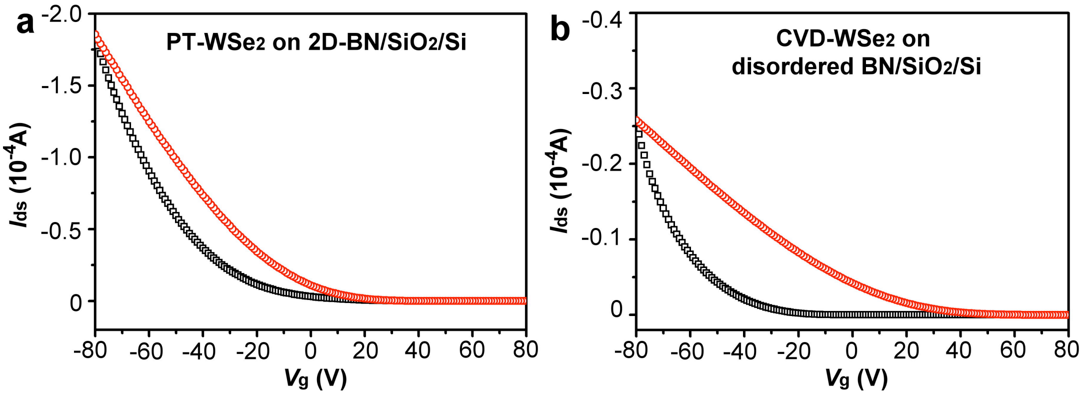

Supplementary Fig. 27. Transfer curves (V_{ds} = −2 V) of **a,** a PT-WSe_2 FET device on 2D-BN/SiO_2/Si and **b,** a CVD-WSe_2 FET device on disordered BN/SiO_2/Si. The black curve is obtained when V_g sweeps from 80 to −80 V, and the red curve is obtained when V_g sweeps from −80 to 80 V.

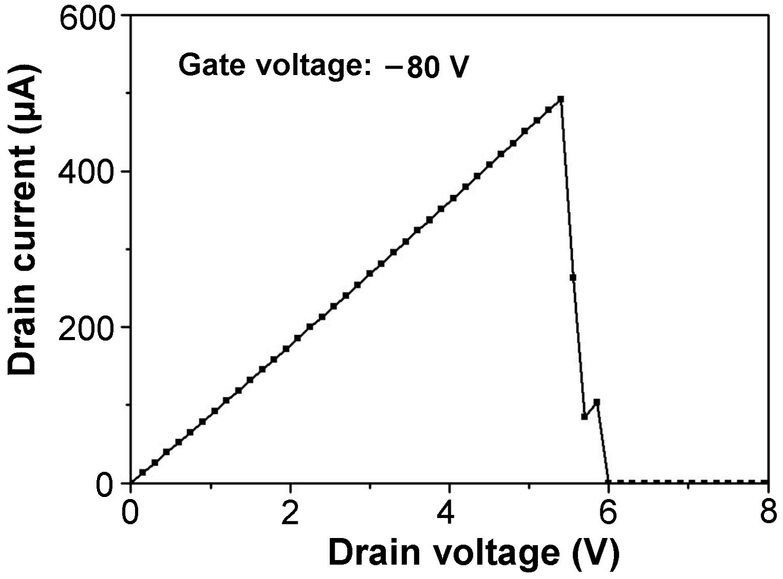

Supplementary Fig. 28. The *I-V* curve of the current breakdown of a CVD-WSe_2 FET device on 2D-BN/SiO_2/Si.

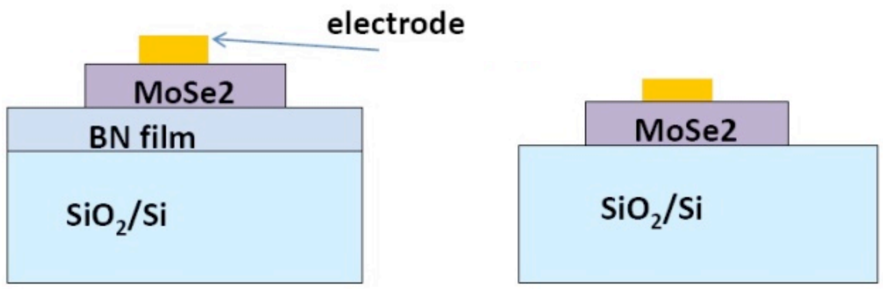

Supplementary Fig. 29. Side-view illustration of devices for differential 3ω measurement.

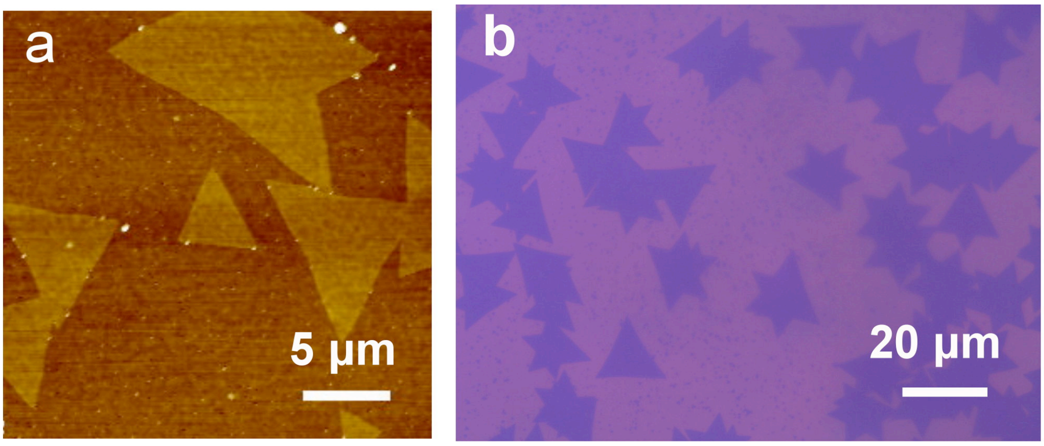

Supplementary Fig. 30. a, AFM and **b,** optical images of CVD-$MoSe_2$ on 2D-BN/SiO_2/Si. The scale bars are 5 μm in **a** and 20 μm in **b**.

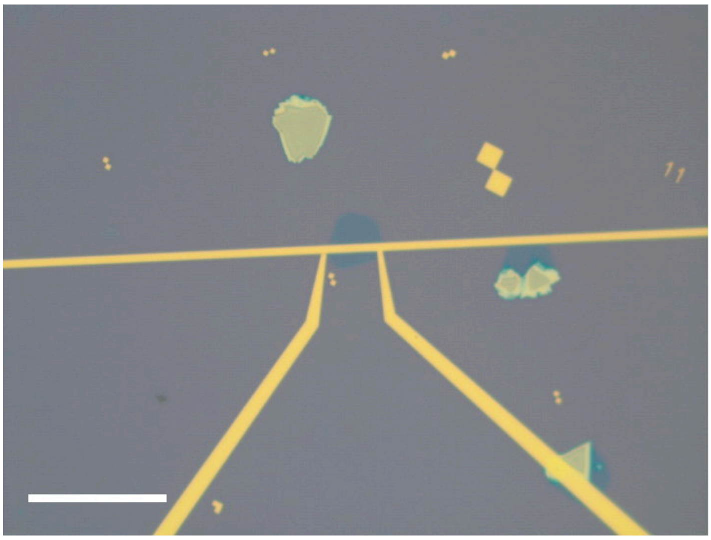

Supplementary Fig. 31. Optical image of a CVD-$MoSe_2$ device on 2D-BN/SiO_2/Si for differential 3ω measurement before plasma etching. The scale bar is 40 μm.

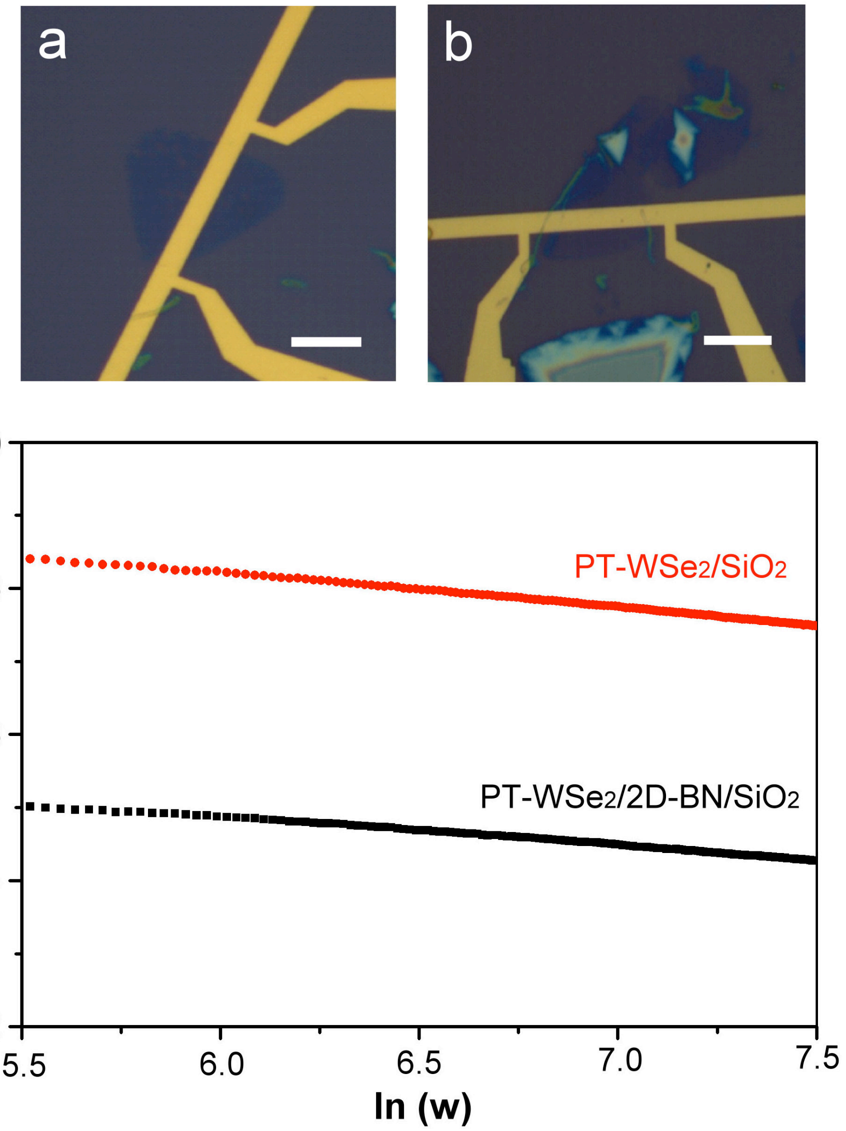

Supplementary Fig. 32. Optical image of a device of PT-WSe_2 on **a,** 2D-BN/SiO_2/Si and **b,** SiO_2/Si for differential 3ω measurement before plasma etching. **c,** $T_{2\omega}$ versus ln ω for PT-WSe_2/2D-BN/SiO_2 interface and PT-WSe_2/SiO_2 interface. The scale bars are 10 μm.

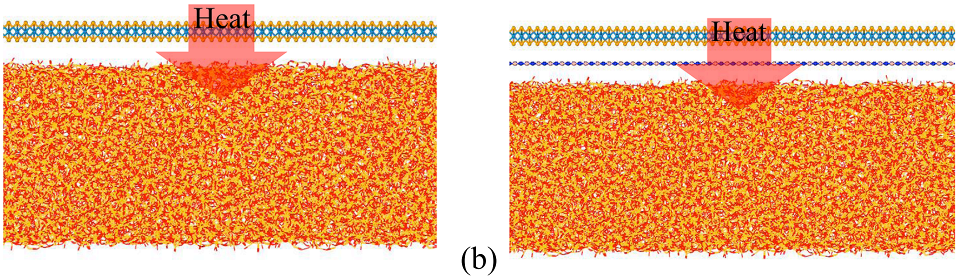

(a) (b)

Supplementary Fig. 33. Schematic figure of MD simulation. The simulation setup for **a,** WSe_2/SiO_2 and **b,** WSe_2/2D-BN/SiO_2 hybrid systems.

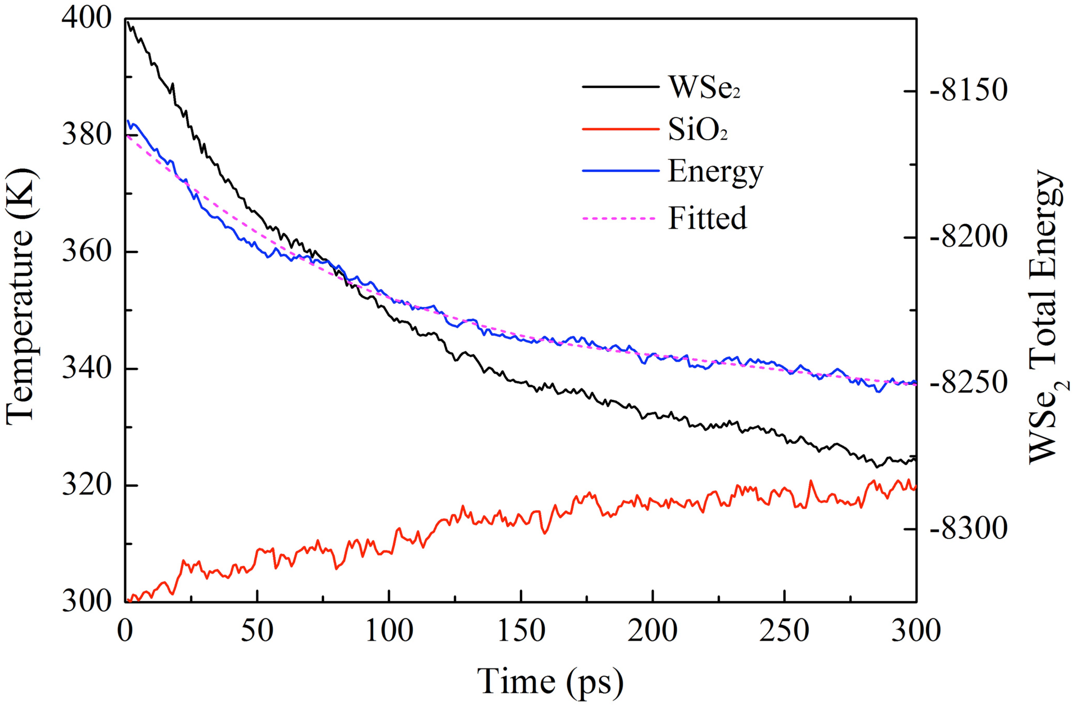

Supplementary Fig. 34. Energy fitting of supported WSe_2 layer for thermal resistance calculations. The temperature of WSe_2 and SiO_2, and total energy of WSe_2 layer are recorded from MD calculations

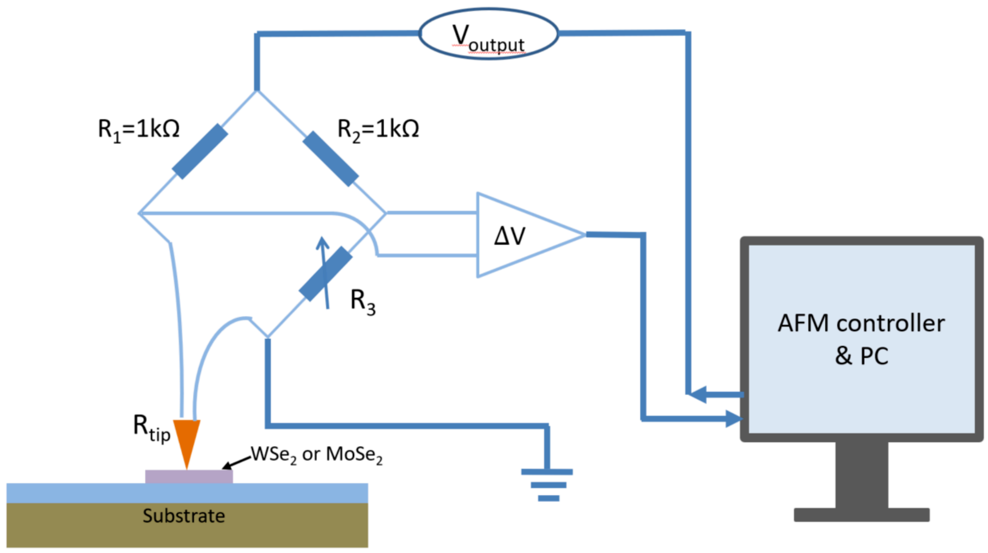

Supplementary Fig. 35. The schematic of the SThM measurement.

Supplementary References

1. El-Yado uni, A., Soltani, A., Boudrioua, A., Thevenin, P., Bath, A. & Loulergue, J. C. Investigation of optical and electro optical properties of hexagonal boron nitride thin films deposited by PECVD technique. *Opt. Mater.* **17,** 319−322 (2001).

2. Carren˜o, M. N. P., Bottecchia, J. P. & Pereyra, I. Low temperature plasma enhanced chemical vapour deposition boron nitride. *Thin Solid Films* **308-309,** 219–222 (1997).

3. Vilcarromero, J., Carreno, M. N. P. & Pereyra, I. Mechanical properties of boron nitride thin films obtained by RF-PECVD at low temperatures. *Thin Solid Films* **373,** 273−276 (2000).

4. Battiston, G. A., Berto, D., Convertino, A., Emiliani, D., Figueras, A., Gerbasi, R. & Viticoli, S. PECVD of *h*-BN and c-BN films from boranedimethylamine as a single source precursor. *Electrochimica Acta* **50,** 4600–4604 (2005).

5. Behura, S., Nguyen, P., Che, S., Debbarma, R. & Berry, V. Large-area, transfer-free, oxide-assisted synthesis of hexagonal boron nitride films and their heterostructures with MoS_2 and WS_2. *J. Am. Chem. Soc.* **137,** 13060−13065 (2015).

6. Jang, A-R., Hong, S., Hyun, C., Yoon, S. I., Kim, G., *et al.* Wafer-scale and wrinkle-free epitaxial growth of single-orientated multilayer hexagonal boron nitride on sapphire. *Nano Lett.* **16,** 3360−3366.

7. Pierson, H. O. Boron nitride composites by chemical vapor deposition. *J. Compos. Mater.* **9,** 228–240 (1975).

8. Liu, D., Yang, W., Zhang, L., Zhang, J., Meng, J., Yang, R., Zhang, G. Y. & Shi, D. X. Two-step growth of graphene with separate controlling nucleation and edge growth directly on SiO_2 substrates. *Carbon* **72,** 387–392 (2014).

9. Wei, D., Lu, Y., Han, C., *et al.* Critical crystal growth of graphene on dielectric substrates at low temperature for electronic devices. *Angew. Chem. Int. Ed.* **125,** 14371−14376. (2013).

10. Reinke, P., Oelhafen, P., Feldermann, H., Ronning, C. & Hofsass, H. Hydrogen-plasma etching of ion beam deposited c-BN films: An in situ investigation of the surface with electron spectroscopy. *J. Appl. Phys.* **88,** 5597−5604 (2000).

11. Schaffnit, C., Thomas, L. & Rossi, F. Etching of boron nitride in radio frequency plasmas. *J. Vac. Sci. Technol. A* **15,** 2816−2819 (1997).

12. Zeng, H., Zhi, C., Zhang, Z., Wei, X., Wang, X., Guo, W., Bando, Y. & Golberg, D. "White graphenes": boron nitride nanoribbons via boron nitride nanotube unwrapping. *Nano Lett.* **10,** 5049−5055 (2010).

13. Mukherjee, R. & Bhowmick, S. Edge stabilities of hexagonal boron nitride nanoribbons: a first-principles study. *J. Chem. Theory Comput.* **7,** 720−724 (2011).

14. Kim, D. Y., Han, N., Jeong, H., Kim, J., Hwang, S., Song, K., Choi, S.-Y. & Kim, J. K. Pressure-dependent growth of wafer-scale few-layer h-BN by metal-organic chemical vapour deposition. *Cryst. Growth Des.* **17,** 2569−2575 (2017).

15. Nemanich, R. J., Solin, S. A. & Martin, R. M., Light scattering study of boron nitride microcrystals. *Phys. Rev. B* **23,** 6348−6356 (1981).

16. Perdew, J. P., Burke, K., Ernzerhof, M. Generalized gradient approximation made simple. *Phys. Rev. Lett.* **77,** 3865−3868 (1996).

17. Kresse, G., Furthmuller, J. Efficient iterative schemes for ab initio total-energy calculations using a plane-wave basis set. *Phys. Rev. B* **54,** 11169−11186 (1996).

18. Blochl, P. E. Projector augmented-wave method. *Phys. Rev. B* **50,** 17953−17979 (1994).

19. Beomjin, P., Kyunghun, K. Anomalous Ambipolar Transport of Organic Semiconducting Crystals via Control of Molecular Packing Structures. *ACS Appl. Mater. Interfaces* **9,** 27839−27846 (2017).

20. Gala, F., Zollo, G. Dielectric Properties of Self-Assembled Monolayer Coatings on a (111) Silicon Surface. *J. Phys. Chem. C* **119,** 7264−7274 (2015).

21. Plimpton, S. Fast parallel algorithms for short-range molecular dynamics. *J. Comput. Phys.* **117,** 1−19 (1995).

22. Munetoh, S., Motooka, T., Moriguchi, K., Shintani, A. Interatomic potential for Si–O systems using Tersoff parameterization. *Comput. Mater. Sci.* **39,** 334−339 (2007).

23. Lindsay, L., Broido, D. A. Enhanced thermal conductivity and isotope effect in single-layer hexagonal boron nitride. *Phys. Rev. B* **84,** 155421 (2011).

24. Shen, M., Keblinski, P. Ballistic vs. diffusive heat transfer across nanoscopic films of layered crystals. *J. Appl. Phys.* **115,** 144310 (2014).

25. Rappe, A. K., Casewit, C. J., Colwell, K. S., Goddard, W. A., Skiff, W. M. UFF, a full periodic table force field for molecular mechanics and molecular dynamics simulations. *J. Am. Chem. Soc.* **114,** 10024−10035 (1992).

26. Gundrum, B. C., Cahill, D. G., Averback, R. S. Thermal conductance of metal-metal interfaces. *Phys. Rev. B* **72,** 245426 (2005).

27. Hong, Y., Li, L., Zeng, X. C., Zhang, J. Tuning thermal contact conductance at graphene–copper interface via surface nanoengineering. *Nanoscale* **7,** 6286−6294 (2015).

28. Zhang, J., Hong, Y., Tong, Z., Xiao, Z., Bao, H., Yue, Y. Molecular dynamics study of interfacial thermal transport between silicene and substrates. *Phys. Chem. Chem. Phys.* **17,** 23704−23710 (2015).
